# Promiscuous targeting of bromodomains by bromosporine identifies BET proteins as master regulators of primary transcription response in leukemia

**DOI:** 10.1126/sciadv.1600760

**Published:** 2016-10-12

**Authors:** Sarah Picaud, Katharina Leonards, Jean-Philippe Lambert, Oliver Dovey, Christopher Wells, Oleg Fedorov, Octovia Monteiro, Takao Fujisawa, Chen-Yi Wang, Hannah Lingard, Cynthia Tallant, Nikzad Nikbin, Lucie Guetzoyan, Richard Ingham, Steven V. Ley, Paul Brennan, Susanne Muller, Anastasia Samsonova, Anne-Claude Gingras, Juerg Schwaller, George Vassiliou, Stefan Knapp, Panagis Filippakopoulos

**Affiliations:** 1 Structural Genomics Consortium, University of Oxford, Old Road Campus Research Building, Roosevelt Drive, Oxford OX3 7DQ, U.K.; 2 Laboratory of Childhood Leukemia, Department of Biomedicine, University of Basel and Basel University Children’s Hospital, Hebelstrasse 20, CH-4031 Basel, Switzerland.; 3 Lunenfeld-Tanenbaum Research Institute, Sinai Health System, Toronto, Ontario, Canada.; 4 Wellcome Trust Sanger Institute, Wellcome Trust Genome Campus, Hinxton, Cambridge CB10 1SA, U.K.; 5 Target Discovery Institute, University of Oxford, Oxford OX3 7FZ, U.K.; 6 Ludwig Institute for Cancer Research, University of Oxford, Oxford OX3 7DQ, U.K.; 7 Department of Chemistry, University of Cambridge, Lensfield Road, Cambridge CB2 1EW, U.K.; 8 Alzheimer’s Research UK Oxford, Nuffield Department of Medicine Research Building, University of Oxford, Oxford OX3 7FZ, U.K.; 9 Department of Oncology, CRUK/MRC Oxford Institute for Radiation Oncology, University of Oxford, Oxford OX3 7DQ, U.K.; 10 Department of Molecular Genetics, University of Toronto, Toronto, Ontario, Canada.; 11 Department of Haematology, Cambridge University Hospitals NHS Trust, Cambridge CB2 0QQ, U.K.; 12 Department of Haematology, University of Cambridge, Cambridge Biomedical Campus, Cambridge CB2 0XY, U.K.; 13 Institute for Pharmaceutical Chemistry and Buchmann Institute for Life Sciences, Goethe University, Max-von Laue Str. 9, 60438 Frankfurt am Main, Germany.

**Keywords:** Bromodomains, inhibition, epigenetics, leukemias, BET

## Abstract

Bromodomains (BRDs) have emerged as compelling targets for cancer therapy. The development of selective and potent BET (bromo and extra-terminal) inhibitors and their significant activity in diverse tumor models have rapidly translated into clinical studies and have motivated drug development efforts targeting non-BET BRDs. However, the complex multidomain/subunit architecture of BRD protein complexes complicates predictions of the consequences of their pharmacological targeting. To address this issue, we developed a promiscuous BRD inhibitor [bromosporine (BSP)] that broadly targets BRDs (including BETs) with nanomolar affinity, creating a tool for the identification of cellular processes and diseases where BRDs have a regulatory function. As a proof of principle, we studied the effects of BSP on leukemic cell lines known to be sensitive to BET inhibition and found, as expected, strong antiproliferative activity. Comparison of the modulation of transcriptional profiles by BSP after a short exposure to the inhibitor resulted in a BET inhibitor signature but no significant additional changes in transcription that could account for inhibition of other BRDs. Thus, nonselective targeting of BRDs identified BETs, but not other BRDs, as master regulators of context-dependent primary transcription response.

## Teaser

BET bromodomains are unique targeting modules that mediate primary transcription response.

## INTRODUCTION

Bromodomains (BRDs) are acetyl-lysine–dependent protein interaction modules that play a pivotal role in chromatin biology and control of gene expression. The human BRD family comprises 61 diverse domains that are present in mainly nuclear proteins. They often act as scaffolding proteins but may also have catalytic functions, such as adenosine triphosphatase–dependent helicase, histone acetyl, or methyl transferase activities (*1*). Acetyl-lysine recognition is mediated by a binding cleft formed by four canonical helices (αZ, αA, αB, and αC) and two connecting loop regions (ZA and BC loops). This interaction site is highly druggable, enabling the development of potent protein interaction inhibitors (*2*, *3*). BRD-containing proteins have been linked to a diversity of diseases, in particular to cancer (*2*, *4*). The first inhibitors developed to target BET (bromo and extra-terminal) proteins showed potent activity not only in cancers that are dependent on chromosomal rearrangements involving BETs but also in other diverse cancer types (*5*, *6*). This unexpected finding has been rationalized by the specific transcriptional down-regulation of growth-promoting and antiapoptotic genes, including key oncogenes, such as *c-Myc* (*6*). The strong growth-inhibiting properties of BET inhibitors therefore rapidly translated into clinical studies that aim to examine their efficacy in oncology (*2*).

The success of BET inhibitors led to the development of programs targeting other BRD proteins, and a number of potent and selective chemical probes have now been published (*5*, *7*–*12*). However, to date, few phenotypic consequences of the inhibition of non-BET BRDs have been reported. To evaluate the benefits of targeting other BRDs, we developed a promiscuous BRD inhibitor with nanomolar potency for 13 BRDs and low micromolar activity for 12 additional BRDs. In analogy with the nonspecific kinase inhibitor staurosporine (*13*, *14*), we named this promiscuous inhibitor bromosporine (BSP). We evaluated the consequences of BSP on transcription in leukemic cell lines, a cancer type that has been studied well using BET inhibitors (*6*). We found that BSP had effects on cell proliferation and clonogenic growth that were similar to those of the pan-BET inhibitor (+)-JQ1 (henceforth JQ1). Genome-wide transcriptional analysis using Illumina microarrays revealed a pronounced BET signature after a short exposure to BSP. In agreement with these data, the selective inhibitors of non-BET BRDs showed only negligible effects on gene transcription, suggesting that BETs—and not any of the other BRDs inhibited by BSP—are dominant mediators of primary transcription response in leukemia. We believe that BSP, similar to staurosporine, will be a versatile tool for studying protein acetylation in chemical biology and will inspire the development of selective BRD inhibitors using related scaffolds.

For the design of BSP, we analyzed a comprehensive collection of BRD structures and complexes of BRDs with histone peptides (*1*, *15*–*20*). This analysis revealed similar binding modes of histone-derived peptides across different BRD structural classes. A channel formed by the ZA loop and helix A is present in most BRD structures, but this pocket is rarely occupied by peptidic histone ligands (fig. S1A). We therefore hypothesized that BRD inhibitors with broad activity against BRDs could target this conserved groove because it offers little possibility for sequence-specific peptide binding while enhancing inhibitor affinity.

**Fig. 1:**
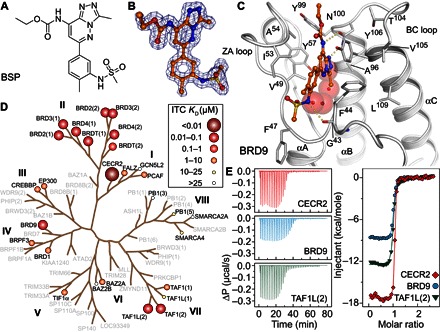
BSP is a pan-BRD inhibitor in vitro. (**A**) Triazolopyridazine scaffold of BSP. (**B**) 2*F*_o_ − *F*_c_ map of BSP bound to BRD4(1) contoured at 2σ. (**C**) Complex of BSP with the BRD of BRD9. The compound adopts an acetyl-lysine mimetic pose within the BRD cavity, initiating interactions with the conserved asparagine (N100). The sulfonamide function initiates contacts with ZA-loop residues (G43), further stabilizing the interaction without displacing any of the structurally conserved water molecules (red spheres). (**D**) BSP binding with low micromolar to nanomolar affinity to most structural classes within the human BRD family. Dissociation constants (*K*_D_) measured in-solution using ITC are displayed on the human BRD tree as spheres (size and color as indicated in the inset). BRD structural classes are annotated with roman numerals. (**E**) Overlay of ITC measurements of typically strong BSP interactions with the BRDs of CECR2, BRD9, and TAF1L(2). Raw injection heats for the titrations of proteins into solutions of BSP are shown on the left panels. The right panel shows the normalized binding enthalpies corrected for the heat of protein dilution as a function of binding site saturation (symbols as indicated in the figure). Solid lines represent a nonlinear least-squares fit using a single-site binding model. All titrations were carried out in 50 mM Hepes buffer (pH 7.5; 25°C) and 150 mM NaCl at 15°C while stirring at 1000 rpm.

Expanding on our previous work on nonselective inhibitors based on a tricyclic chemotype (*21*), we selected a similar triazolopyridazine dicyclic core scaffold to explore the development of potent promiscuous inhibitors. We rationalized that scaffold expansion toward the identified binding groove and toward the BC loop would help avoid subfamily-unique features, such as the WPF [tryptophan (W)–proline (P)–phenylalanine (F)] shelf that would confer selectivity toward the BET family (fig. S1B). Compounds developed as part of a small focused library of dicyclic chemotypes, modified at two positions (fig. S1C), exhibited broad activity in a thermal stability assay against a diverse set of human BRD targets that comprised representative members of all BRD structural subfamilies (fig. S2A). Several optimization cycles led to a compound that potently inhibited most BRDs, which we named “BSP” and selected for further characterization (Fig. 1A, fig. S2B, and table S1).

## RESULTS

### BSP is a potent pan-BRD inhibitor in vitro

To confirm the promiscuous targeting of the human BRD family by BSP, we screened BSP with biolayer interferometry (BLI) against a panel of 42 recombinant biotinylated BRDs that covered all structural families (fig. S2C). In agreement with our thermal melt data, BSP showed broad activity against the BRDs of diverse families. To ascertain the predicted promiscuous binding mode, we determined high-resolution crystal structures with different human BRDs. In all cases, BSP was resolved well in the high-resolution structures (Fig. 1B) and inserted into the acetyl-lysine binding cavity of each BRD module. The binding mode was conserved between BRD9 (Fig. 1C), the first BRD of BRD4 [BRD4(1); fig. S2D], and the second BRD of TAF1L [TAF1L(2); fig. S2E]. In all cases, BSP exhibited a common binding mode, whereby it engaged the conserved asparagine [N100 in BRD9, N140 in BRD4(1), and N1602 in TAF1L(2)] while extending its sulfonamide substituent toward the front of the ZA-loop binding cavity. The excellent shape complementarity with diverse acetyl-lysine binding sites explained its high binding potency, which we further characterized in solution by isothermal titration calorimetry (ITC) (Fig. 1, D and E). For several target subfamilies, BSP represents the most potent inhibitor developed to date (Fig. 1D and Table 1). BSP exhibited affinity toward previously untargeted BRDs, such as TAF1L(2) (*K*_D_ = 43 nM), and provided a novel chemical starting point for BRDs that had been previously targeted with only very weak compounds, such as P300/CBP-associated factor (PCAF) (BSP *K*_D_ = 4.7 μM).

**Table 1:** ITC of human BRDs with BSP. Titrations were carried out in 50 mM Hepes buffer (pH 7.5; 25°C) and 150 mM NaCl at 15°C while stirring at 1000 rpm. Proteins were titrated into the ligand solution (reverse titration). Values are means ± SD.

**Protein**	**[P] (μM)**	**[L] (μM)**	***K*_D_ (nM)**	**Δ*H*^obs^ (kcal/mol)**	***N***	***T*Δ*S* (kcal/mol)**	**Δ*G* (kcal/mol)**
BAZ2A	433	15	3,745 ± 291	−3.04 ± 0.095	1.05 ± 0.025	4.12	−7.16
BAZ2B	607	15	Weak binding				
BRD1	440	13	1,653 ± 66	−7.32 ± 0.078	1.01 ± 0.008	0.30	−7.62
BRD2(1)	271	25	97.1 ± 6.7	−7.90 ± 0.034	1.00 ± 0.003	1.34	−9.25
BRD2(2)	235	25	50.3 ± 5.0	−5.46 ± 0.028	1.10 ± 0.003	4.18	−9.64
BRD3(1)	275	20	91.7 ± 5.3	−10.02 ± 0.039	1.00 ± 0.002	−0.95	−9.08
BRD3(2)	305	25	50.0 ± 4.7	−8.62 ± 0.041	1.11 ± 0.003	1.01	−9.63
BRD4(1)	258	20	41.8 ± 2.8	−11.09 ± 0.038	0.94 ± 0.002	−1.36	−9.73
BRD4(2)	270	20	39.7 ± 2.2	−6.60 ± 0.018	0.94 ± 0.001	3.17	−9.77
BRDT(1)	228	20	40.2 ± 2.8	−13.16 ± 0.047	1.02 ± 0.002	−3.40	−9.76
BRDT(2)	271	20	172.1 ± 10.6	−5.61 ± 0.028	1.00 ± 0.003	3.31	−8.92
BRD9	251	25	41.7 ± 3.8	−8.75 ± 0.039	1.00 ± 0.002	0.98	−9.73
BRPF1B	406	20	311.5 ± 11.2	−6.12 ± 0.021	1.00 ± 0.003	2.45	−8.57
BRPF3	400	15	8,621 ± 381	−4.20 ± 0.123	1.08 ± 0.025	2.48	−6.68
CECR2	202	16	8.0 ± 1.0	−17.28 ± 0.062	1.04 ± 0.002	−6.60	−10.68
CREBBP	617	25	1,524 ± 116	−2.91 ± 0.041	1.03 ± 0.011	4.72	−7.64
EP300	460	15	7,194 ± 501	−5.65 ± 0.265	0.97 ± 0.036	1.13	−6.78
BPTF	230	15	1,887 ± 53	−10.09 ± 0.074	1.07 ± 0.005	−3.28	−6.80
GCN5L2	336	15	Weak binding				
PB1(3)	389	15	Weak binding				
PB1(5)	604	15	14,225 ± 802	−3.19 ± 0.183	1.09 ± 0.053	3.20	−6.39
PCAF	610	13	4,762 ± 459	−7.85 ± 0.454	0.95 ± 0.044	−0.83	−7.02
SMARCA2	222	25	Weak binding				
SMARCA4	400	13	19,685 ± 838	−9.05 ± 0.580	0.99 ± 0.055	−2.84	−6.21
TAF1(1)	460	23	5,525 ± 199	−3.34 ± 0.046	0.98 ± 0.010	3.60	−6.94
TAF1(2)	230	20	16.6 ± 2.7	−14.05 ± 0.095	0.98 ± 0.003	−3.80	−10.25
TAF1L(1)	610	13	25,000 ± 2,027	−4.78 ± 0.778	1.00 ± 0.146	1.29	−6.07
TAF1L(2)	250	20	42.7 ± 4.4	−12.48 ± 0.069	0.99 ± 0.003	−2.76	−9.72
TIF1A	400	15	8,475 ± 431	−2.61 ± 0.088	1.09 ± 0.029	4.06	−6.67

### BSP engages its target BRDs in cells

**Fig. 2:**
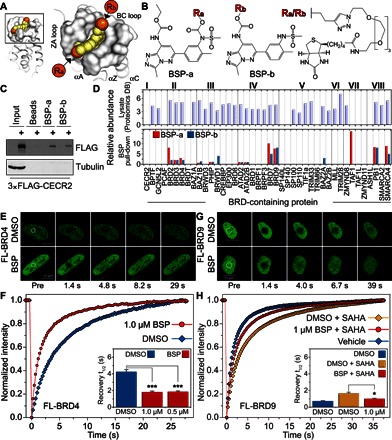
BSP engages its target BRDs in cells. (**A**) BSP binds to the BRD acetyl-lysine cavity, allowing for further functionalization toward the front channel within the ZA loop (Ra vector annotated in orange) or the back of the pocket (Rb vector annotated in orange). The vectors are shown in the complex of BSP with BRD4(1). (**B**) Two variants of biotinylated BSP (BSP-a and BSP-b) were prepared to explore binding to human BRDs in cells by pull-down experiments. (**C**) Biotinylated BSP (BSP-a or BSP-b) immobilized on magnetic beads was used to pull down human CECR2 from Flp-In T-REx HEK293 cells stably expressing 3×FLAG CECR2. The protein captured from whole-cell lysate was identified using anti-FLAG. (**D**) Cell lysate from HEK293T cells was incubated with biotinylated BSP (BSP-a or BSP-b) immobilized on magnetic streptavidin beads in the presence or absence of 30 nmol of BSP for 2 hours at 4°C. After pull-down and tryptic digestion with trypsin, proteins were identified in a TripleTOF 5600 mass spectrometer. (Top) Normalized abundance of each BRD-containing protein in HEK293 cells (data from Proteomics DB; https://www.proteomicsdb.org/). (Bottom) Ratio of peptide to peptide abundance in the presence and absence of competing BSP, shown as a bar graph. BRD families are annotated with roman numerals. (**E**) FRAP evaluation of full-length GFP-tagged BRD4 dissociation from chromatin in U2OS cells. Nuclei of DMSO-treated (top) or BSP-treated (1 μM; bottom) cells. Target regions of photobleaching are indicated with a white circle. Scale bar, 10 μm. FL-BRD4, full-length BRD4; FL-BRD9, full-length BRD9. (**F**) Quantitative comparison of time to half-maximal fluorescence recovery for BRD4 FRAP studies using BSP (red bars) as a function of ligand concentration. (**G**) FRAP evaluation of full-length GFP-tagged BRD9 dissociation from chromatin in U2OS cells. Nuclei of DMSO-treated (top) or BSP-treated (1 μM; bottom) cells in the presence of 10 μM SAHA (added to increase the experimental window). Target regions of photobleaching are indicated with a white circle. Scale bars, 10 μm. (**H**) Quantitative comparison of time to half-maximal fluorescence recovery for BRD9 FRAP studies using BSP (red bars) as a function of ligand concentration. Data in (F) and (H) represent means ± SEM (*n* = 30) and are annotated with *P* values obtained from a two-tailed *t* test (*P < 0.05 and ***P < 0.001).

Next, we were interested to see whether BSP would exhibit similar promiscuous binding to BRD-containing proteins in cells. To address this, we synthesized BSP adducts with biotin tethered by a flexible linker. The versatile pyridazine portion of BSP allowed positioning of a tag that would point away from the central BRD cavity (Fig. 2A), resulting in two biotinylated variants (Fig. 2B), which largely retained the in vitro affinity toward BRDs (Table 2). The biotinylated BSP variants could engage with CECR2 in human embryonic kidney (HEK) 293 cells stably expressing 3×FLAG CECR2 (Fig. 2C). Encouraged by this result, we used the biotin tag to extract and purify BRDs, applying mass spectrometry (MS) together with pull-down experiments using BSP biotin adducts. Although most BRD-containing proteins are expressed in HEK293 cells, we observed enriched binding to most high-affinity in vitro targets of BSP (Fig. 2D and Table 3) and also identified binding partners from larger complexes, such as the SWI/SNF BRD-containing proteins SMARCA2/SMARCA4 and PB1, which were most likely enriched as a result of a tight interaction with the BSP targets BRD7/BRD9 (*22*). We next asked whether BSP directly engages its BRD targets in cells in the acetyl-lysine competitive mode of action suggested by our structural models. We used a fluorescence recovery after photobleaching (FRAP) technique to disrupt the interaction of full-length green fluorescent protein (GFP)–tagged BRD4 (Fig. 2, E and F) or full-length GFP-tagged BRD9 (Fig. 2, G and H) with acetylated chromatin. In both cases, we observed displacement of proteins from chromatin, as evidenced by the fast recovery after bleaching (Fig. 2, F and H). In the case of BRD9, we treated cells with the histone deacetylase inhibitor SAHA (suberoylanilide hydroxamic acid) to increase acetylation levels and to enhance binding (Fig. 2H). For a representative selection of BRDs tested in the presence of BSP, we observed a significant shortening of fluorescence recovery times indicative of inhibition of chromatin-BRD interaction.

**Table 2:** Δ*T*_m_ shifts (°C) of biotinylated BSP (BSP-a and BSP-b) tested against a panel of BET and other diverse BRDs. Compounds (final concentration, 10 μM) were added to the proteins (final concentration, 2 μM); the temperature was increased from 25° to 96°C at a step of 3°C/min; excitation and emission filters for the SYPRO Orange dye were set to 465 and 590 nm; and experiments were performed in triplicate. Values are means ± SD.

**Protein**	**Δ*T*_m_^obs^ (°C)**	
	**BSP-a**	**BSP-b**
BRD2(1)	−7.5 ± 0.7	1.9 ± 0.5
BRD2(2)	−0.4 ± 0.2	3.0 ± 0.2
BRD3(1)	−2.7 ± 0.2	2.3 ± 0.3
BRD3(2)	−3.0 ± 0.1	3.1 ± 0.0
BRD4(1)	−2.7 ± 0.2	3.8 ± 0.3
BRD4(2)	−5.1 ± 0.0	1.8 ± 0.3
BRDT(1)	−5.0 ± 0.1	1.3 ± 0.3
BRDT(2)	0.5 ± 0.1	0.9 ± 0.4
CECR2	4.3 ± 0.0	7.4 ± 0.1
CREBBP	−3.1 ± 0.6	0.1 ± 0.4
TAF1(2)	−12.4 ± 0.1	1.9 ± 0.1
TAF1L(2)	0.2 ± 0.1	2.9 ± 0.4

**Table 3:** Relative abundance of BRD-containing proteins in HEK293 cells (data taken from Proteomics DB; https://www.proteomicsdb.org/). Pull-down of human BRD-containing proteins with biotinylated BSP (BSP-a and BSP-b), followed by competitive elution with BSP and MS, resulted in enrichment of BSP-targeted BRDs.

**Protein**	**Relative peptide abundance**			**Protein**	**Relative peptide abundance**		
	**293**	**BSP-a**	**BSP-b**		**293**	**BSP-a**	**BSP-b**
CECR2	—	—	—	BRD7	4.51	10	5
BPTF	4.31	—	—	BRD9	4.57	7.6	8
GCN5L2	3.75	—	—	SP140L	—	—	—
PCAF	—	—	—	SP140	—	—	—
BRD2	5.59	8	2	SP100	3.43	—	—
BRD3	5.04	2	2	SP110	2.71	—	—
BRD4	5.17	3	2	TIF1a	4.91	—	—
BRDT	—	—	—	TRIM33	5.34	—	—
BAZ1A	5.19	—	—	TRIM66	—	—	—
BAZ1B	5.63	0.64	1.33	BAZ2A	4.25	—	3
BRWD3	3.61	—	—	BAZ2B	3.21	—	—
PHIP	5.05	1.09	1.15	MLL	3.67	—	—
BRWD1	3.24	—	4	TRIM28	7.05	—	—
CREBBP	4.61	—	—	ZMYND8	4.4	—	—
EP300	4.87	—	—	TAF1	—	16	—
BRD8	4.48	—	—	TAF1L	—	—	—
ATAD2	4.65	0.98	0.87	ZMYND11	—	—	—
ATAD2B	3.68	0.90	1	ASH1L	—	—	—
BRD1	3.48	—	—	PB1	5.23	8.28	8
BRPF1	3.6	—	—	SMARCA2	4.44	—	—
BRPF3	3.42	—	—	SMARCA4	5.53	8.8	5

### BSP inhibits growth of cancer cell lines

**Fig. 3:**
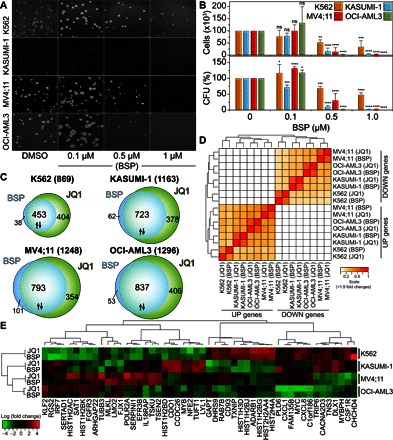
BSP inhibits growth in leukemia cell lines. (**A**) BSP inhibits clonogenic growth in leukemia cell lines. K562, KASUMI-1, MV4;11, and OCI-AML3 in methylcellulose were treated with vehicle (DMSO) or BSP (0.1, 0.5, or 1 μM) (*n* = 4). (**B**) Colony formation assay in K562, KASUMI-1, MV4;11, and OCI-AML3 cells using 0.1, 0.5, or 1.0 μM BSP (top) and the number of cells counted after treatment of cells with BSP for 6 to 10 days (*n* = 4) (bottom). CFU, colony-forming units; ns, not significant. (**C**) Similarity comparison of significantly expressed genes (*P* < 0.001 and fold change > 1.5) in the four cell lines after drug treatment. The heat map represents the intersect matrix for all pairwise comparisons (cell lines and treatments) using euclidean distances and complete linkage after transformation of the intersect counts into similarity Jaccard measures. (**D**) Venn diagrams showing overlap of the top statistically significant (Benjamini-Hochberg adjusted *P* < 0.001) genes (up- or down-regulated with a fold change of >1.5) differentially expressed by BSP or the pan-BET inhibitor JQ1 in four leukemia cell lines (K562, KASUMI-1, OCI-AML3, and MV4;11) after 8 hours of treatment with the inhibitor (0.5 μM) (top) and breakdown of the expression in terms of up- and down-regulated genes for each cell line (bottom). (**E**) Heat map of log fold changes in the expression of the top 50 statistically significant genes in the four cell lines tested, identified using Benjamini-Hochberg adjusted *P* < 0.001. Data in (B) represent means ± SEM (*n* = 4) and are annotated with *P* values obtained from a two-tailed *t* test (*P < 0.05, **P < 0.01, ***P < 0.001, and ****P < 0.0001).

To further assess the effects of BSP on cellular systems, we profiled this inhibitor against the National Cancer Institute (NCI) panel of cancer cell lines (NCI-60). BSP exhibited strong growth inhibition in all cancer types (fig. S3, A to I), including leukemia (fig. S3D). We were particularly interested in this cancer type because we previously observed strong inhibition of the growth of leukemia cell lines when we used the pan-BET inhibitors JQ1 and PFI-1 (*5*, *7*). We therefore investigated the ability of BSP to inhibit the clonogenic growth and proliferation of two acute myeloid leukemia (AML) cell lines (MV4;11 and KASUMI-1), the hyperdiploid AML line OCI-AML3, and the BCR-ABL–positive chronic myeloid leukemia (CML) cell line K562, and we observed growth inhibition in the concentration range 100 to 500 nM (Fig. 3A and fig. S3J). Colony formation by the cells was decreased at 500 nM BSP and severely inhibited at 1 μM BSP (Fig. 3B). Given the potent inhibition observed in colony formation, we also compared the effect of BSP on clonogenic growth to the effect of the pan-BET inhibitor JQ1, which was previously shown to potently and effectively suppress proliferation in AML (*6*). K562 cells were relatively resistant to BSP, similar to JQ1, whereas we measured nanomolar median inhibitory concentration (IC_50_) values for both inhibitors in MV4;11 and KASUMI-1 cells (fig. S4A). In summary, our data showed that BSP potently inhibited colony formation and proliferation of leukemic cell lines with an efficacy that was slightly weaker than that of pan-BET inhibitors, such as JQ1, in agreement with its comparable in vitro potency toward BET family members.

### BSP modulates transcription in leukemic cell lines

**Fig. 4:**
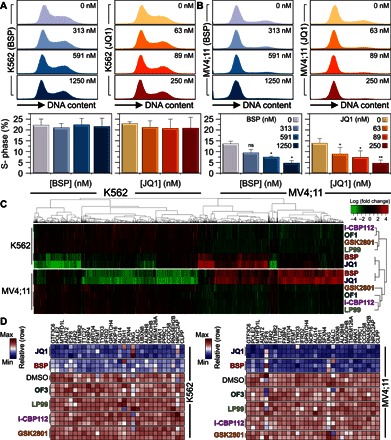
Comparison of the effects of BET inhibition on cell cycle and transcription in leukemias. (**A** and **B**) Cell cycle analysis of BSP (blue scale) and JQ1 (red scale) inhibition in resistant cells (A; K562) or sensitive cells (B; MV4;11) after 48 hours of treatment with inhibitors (amounts as indicated in the inset). The quantification given below each graph depicts the percent S-phase content measured under each condition, indicating a clear arrest in the sensitive line with minor effects on the resistant line. Data are means ± SEM (*n* = 3) and are annotated with *P* values obtained from analysis of variance (ANOVA) followed by Dunnett’s test (*P < 0.05 and **P < 0.01). (**C**) Heat map of fold changes (expressed in log_2_ scale as indicated in the inset) in the top 1000 significantly differentially expressed genes (Benjamini-Hochberg adjusted *P* < 0.001) in K562 cells (top) and MV4;11 cells (bottom) after 6 hours of treatment with selective BRD inhibitors or DMSO. The effects of JQ1 and BSP are very similar and much stronger than the effects of any other compounds targeting non-BET BRDs. (**D**) A published set of genes constituting a “JQ1 signature” is only attenuated by BSP and JQ1 in K562 cells (left) and MV4;11 cells (right) after 6 hours of treatment with a series of inhibitors (500 nM). The heat map depicts row-normalized values for gene expression, as indicated in the inset.

To better understand the contribution of BRDs to the transcriptional landscape in leukemia, we compared the primary effects on transcription after a short exposure to BSP or JQ1 in the three sensitive AML cell lines (MV4;11, KASUMI-1, and OCI-AML3) and in the less sensitive CML cell line (K562). Principal components analysis revealed that gene expression data sets of each cell line clustered together with no obvious outliers, validating the quality of the gene expression data (fig. S4B). Genes attenuated by either inhibitor were very similar (Fig. 3C). Pairwise comparison of significantly up- and down-regulated genes (*P* < 0.001 and fold change > 1.5) showed a strong correlation between the two inhibitors, suggesting that BET BRDs may be principally responsible for the observed effect on transcription (Fig. 3D). Both inhibitors resulted in very similar fold changes for the most significantly regulated (*P* < 0.001 and fold change > 1.5) genes in each studied cell line, although the effects of BSP and JQ1 on gene transcription were highly context-dependent (Fig. 3E). In contrast, comparison between sensitive cell lines and the less sensitive K562 cells revealed significant differences in regulated genes. Many genes with key functions in tumorigenesis, such as transcription factors (*GATA1*, *FOXA3*, and *HOXA5*), tyrosine kinases (*CSF1R* and *FES*), and apoptosis regulators (*BCL2* and *BCL6B*), were differentially regulated in highly BET inhibitor–sensitive cell lines (MV4;11 and OCI-AML3) in comparison to the K562 line (fig. S4C). Independent validation of the effects of BSP and JQ1 by quantitative real-time polymerase chain reaction (qRT-PCR) confirmed inhibitor effect in a dose-dependent fashion on the four leukemic cell lines (fig. S4D). Among the most significantly deregulated genes were histone clusters (linker, H2, and H3, including isoforms), which were generally strongly up-regulated in K562 and down-regulated in BSP- and JQ1-sensitive cells (fig. S5A). We suspected that a differential effect on cell cycle may be a principal factor regulating sensitivity to BSP in these cell lines. Transcription of histones is typically amplified by 20- to 30-fold during the G_1_-to-S transition (*23*). We hypothesized that sensitive cells may be arrested before this transition while less sensitive cells are cycling normally, resulting in higher levels of histones. We therefore investigated the effect of BSP on the cell cycle. Although BSP and JQ1 had little effect on cell cycle in the broad range of concentrations tested in the less sensitive K562 cells (Fig. 4A), the sensitive MV4;11 cells were potently affected and exhibited distinct G_1_ arrest with reduced S-phase populations (Fig. 4B).

Despite the differences across cell lines, we were surprised by the high similarity in transcription response caused by both inhibitors and by the degree of the observed changes in gene expression (fig. S5B). Given the small differences in individual genes, we decided to investigate the underlying gene ontologies enriched by each inhibitor in the four cell lines. Surprisingly, between inhibitors, there were differences in the underlying biological processes perturbed (fig. S6) or in the cellular components affected (fig. S7A) within the same line. We also performed a MetaCore analysis based on manually curated ontologies, and we found similar enrichment of pathways or process networks between inhibitors for the same line (fig. S7B and table S2). We next performed gene set enrichment analysis (GSEA), seeking to identify enrichment of similar functions between the four leukemia lines. BSP-treated cells enriched several signatures from the Molecular Signatures Database (MSigDB), including a very strong association with signatures suggesting *c-Myc* down-regulation (figs. S8 and S9).

### BSP exhibits a strong BET-like signature in leukemia

We were intrigued by the strong association with *c-Myc* down-regulation signatures uncovered by GSEA. *c-Myc* transcriptional down-regulation has been recognized as a dominant hallmark of BET inhibition in many different tumor types (*24*), and *c-Myc* reexpression has been recently linked to BET inhibitor resistance (*25*, *26*). Zuber *et al.* (*6*) reported strong transcriptional attenuation of the AML cell line THP1 after a 24-hour treatment with JQ1. With these data, we constructed a gene set signature using genes that were strongly down-regulated in THP1 cells (*P* < 0.001 and fold change < −4) and interrogated our four cell lines with GSEA. Both BSP and JQ1 elicited a strong response and enriched this THP1 signature in all four cell lines (fig. S10, A to D), suggesting that the effect of the transcriptional response conferred by BSP occurs through BET BRDs. To further test this, we explored a small gene set that was previously reported to be regulated by JQ1 in neuroblastoma, multiple myeloma, and AML (*27*), and we found strong enrichment with BSP and JQ1 in all four cell lines (fig. S10, E to H). Our data therefore suggest a dominant transcriptional effect through BET proteins, despite the promiscuous targeting of several diverse BRD families by BSP.

Because BSP inhibits a large number of BRD-containing proteins, we next addressed the relative expression level of these proteins in the cell lines studied. We were surprised to find that most BSP target proteins were expressed across all four cell lines (fig. S11A). We would expect BSP to bind to these proteins, reducing the effective concentration of available BSP in the cells. We therefore investigated the effects of non-BET BRD inhibitors specific for family III [CREBBP/EP300 using I-CBP112 (*28*)], family IV [BRD7/BRD9 using LP99 (*11*) and BRPF1/BRPF3 using OF1 (*12*)], and family V [BAZ2A/BAZ2B using GSK2801 (*9*)] BRD-containing proteins that are also targeted by BSP (fig. S11B). We performed genome-wide expression analysis in the pan-BET inhibitor–sensitive line MV4;11 and in the less sensitive line K562, focusing on the same initial response window of 8 hours. We observed a striking resemblance between BSP and JQ1 compared to all other inhibitors tested, with a clear separation of transcriptional responses in both lines (Fig. 4C). As expected, most of the significant genes attenuated (with *P* < 0.001 and fold change > 1.5) were due to BET BRD inhibition by JQ1; however, a very small subset remained unique to BSP and did not overlap with any of the other inhibitors, suggesting that another BSP target or synergistic inhibition of BETs, in combination with other BRD targets, was responsible for this population. This was also true for some significant genes (*P* < 0.001), with smaller fold changes that did not overlap with transcription responses observed using other BRD inhibitors (fig. S12, A and B). We also noticed that attenuation of the most significantly regulated genes was systematically different between BSP and JQ1 and all other inhibitor classes (fig. S13A). At the gene level, there was a small overlap between inhibitor classes (fig. S13, B and C); however, none of the non-BET inhibitors resulted in attenuation of the previously reported JQ1 signature, which persisted between tissue types (*27*) (Fig. 4D). To assess whether BSP and JQ1 would act synergistically, we performed a cell toxicity study in which eight concentrations of each inhibitor were systematically combined in the BET inhibitor–sensitive (MV4;11) and less sensitive (K562) cell lines (fig. S13D). This study showed that when both inhibitors were combined at concentrations significantly lower than the individual median effective concentration (EC_50_) values of the single compounds, survival of both cell lines was increased, whereas at concentrations slightly below each individual EC_50_ value, we observed synergistic effects of cellular toxicity (fig. S13E).

To obtain insights into time-dependent changes in gene expression caused by pan-BET inhibition, we analyzed previously published data on sensitive AML cells treated with JQ1 to define the transcriptional response conferred by BETs after a long (24 hours) treatment. We performed GSEA against all oncogenic signatures (MSigDB c6-gene set) and constructed distinct directional networks for JQ1-treated THP1 cells, resulting in a complex network of signatures (fig. S14A). GSEA followed by distinct directional network construction of BSP-induced signatures revealed remarkable overlap with many of the late-response JQ1-induced signatures observed in THP1 cells, but also significant enrichment of gene sets associated with early response to BET inhibition, such as down-regulation of the epidermal growth factor receptor/mitogen-activated protein kinase pathway (Fig. 4, B and C), whereas longer exposure was characterized by transcriptional regulation of key transcription factors of the E2F and HOX families.

## DISCUSSION

The study presented here revealed a dominant function of BET BRDs regulating gene transcription, compared to other selective BRD inhibitors or the developed promiscuous BRD inhibitor BSP. Thus, inhibition of non-BET BRDs is not likely to affect short-term gene expression, at least in the cellular systems studied here. Selective inhibitors for the BRDs present in SMARCA4/PB1 showed no significant effects on gene expression but significantly contributed to the regulation of cell-specific gene expression programs during cellular differentiation of trophoblasts and stem cells (*12*). Similarly, inhibition of CREBBP/EP300 BRDs specifically affected differentiation of leukemia-initiating cells after prolonged exposure (*28*). These data suggest that non-BET BRDs may be required for the organization of chromatin structure during cell differentiation. In combination with genome-editing techniques, chemical tools (such as BSP) that target multiple BRDs can help rapidly establish key potential BRD targets suitable for drug development. A recent study demonstrated how CRISPR (clustered regularly interspaced short palindromic repeats)/CAS9 can be used to identify BRD4 as an important drug target that sustains murine AML cells (*29*). BSP represents a versatile promiscuous pan-BRD inhibitor class that has limited off-target effects (fig. S15 and table S3) and can be used as a front-line tool to interrogate the role of the acetylation-dependent reading process in cellular systems, leading to similar observations compared to genetic approaches. Therefore, BSP is a valuable tool for the identification of BRD-dependent cellular processes, helping accelerate cell-based studies in this emerging target area.

## MATERIALS AND METHODS

### Synthesis of BSP

**Scheme 1:**
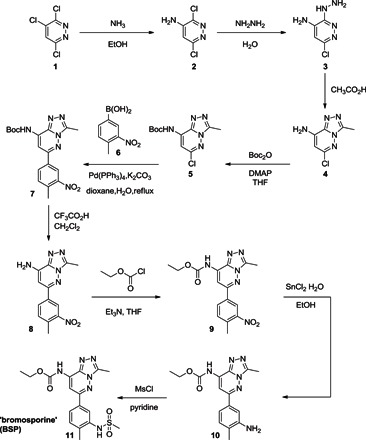
Synthesis of BSP.

BSP [ethyl (3-methyl-6-(4-methyl-3-(methylsulfonamido)phenyl)-[1,2,4]triazolo[4,3-*b*]pyridazin-8-yl)carbamate] was prepared according to Scheme 1.

Intermediate materials were prepared as follows:

3,6-Dichloropyridazin-4-amine (**2**): A stainless steel reaction vessel was charged with 3,4,6-trichloropyridazine (**1**) (1 g, 5.46 mmol) and absolute ethanol saturated with ammonia (40 ml) at 0°C. The vessel was sealed, and the mixture was heated at 125°C for 7 hours. The reaction mixture was evaporated to dryness, and the crude product was recrystallized from water to yield **2** (547 mg, 61%). MS [electrospray ionization (ESI)]: mass/charge ratio (*m*/*z*) for [C_4_H_3_Cl_2_N_3_+H]^+^—calculated, 164 (2× ^35^Cl), 166 (^35^Cl/^37^Cl), and 168 (2× ^37^Cl); found, 163.9, 165.9, and 167.8.

6-Chloro-3-hydrazinylpyridazin-4-amine (**3**): Dichloride **2** (547 mg, 3.33 mmol) and hydrazine (10 ml) were heated to reflux for 3 hours. The mixture was cooled to room temperature, and water (5 ml) was added. The resulting crystalline precipitate was collected, washed with cold water, and dried under reduced pressure to obtain **3** (210 mg, 40%). MS (ESI): *m*/*z* for [C_4_H_6_ClN_5_+H]^+^—calculated, 160 (^35^Cl) and 162 (^37^Cl); found, 159.9 and 161.9.

6-Chloro-3-methyl-[1,2,4]triazolo[4,3-*b*]pyridazin-8-amine (**4**): Hydrazine **3** (210 mg, 1.32 mmol) and acetic acid (3 ml) were heated to reflux for 5 hours. Ice water was added, and the resulting precipitate was collected by filtration. Recrystallization of the crude product from methanol afforded the desired **4** (200 mg, 82%). MS (ESI): *m*/*z* for [C_6_H_6_ClN_5_+H]^+^—calculated, 184 (^35^Cl) and 186 (^37^Cl); found, 184.1 and 186.1.

*tert*-Butyl (6-chloro-3-methyl-[1,2,4]triazolo[4,3-*b*]pyridazin-8-yl)carbamate (**5**): Boc_2_O (7.1 g, 32.9 mol) and 4-dimethylaminopyridine (DMAP) were added to a solution of amine **4** (2 g, 10.9 mmol) in tetrahydrofuran (THF) (60 ml) at 0°C. The mixture was allowed to warm to room temperature, and the reaction progress was monitored by thin-layer chromatography (TLC). Upon completion, the mixture was concentrated under reduced pressure, and the resulting solution was diluted with EtOAc, washed with brine, and dried (Na_2_SO_4_). The solvent was removed in vacuo, and the residue was purified by flash column chromatography (petroleum ether/EtOAc, 1:2) to yield **5** (1.2 g, 40%). MS (ESI): *m*/*z* for [C_11_H_14_ClN_5_O_2_+H]^+^—calculated, 284.1 (^35^Cl) and 286.1 (^37^Cl); found, 284.1 and 285.0.

*tert*-Butyl (3-methyl-6-(4-methyl-3-nitrophenyl)-[1,2,4]triazolo[4,3-*b*]pyridazin-8-yl)carbamate (**7**): Pd(PPh_3_)_4_ (61 mg, 10% eq) and K_2_CO_3_ (182 mg, 1.32 mmol) were added to a mixture of chloride **5** (150 mg, 0.53 mmol) and boronic acid **6** (287 mg, 1.59 mmol) in dioxane/water (5.5 ml, 10:1 v/v), and the resulting mixture was heated at 120°C under Ar. The reaction was monitored by TLC. Upon completion, water was added, and the combined aqueous layers were extracted with dichloromethane (DCM). The organic layers were combined and dried (Na_2_SO_4_). The solvent was removed in vacuo, and the residue was purified by flash column chromatography (DCM/MeOH, 50:1) to give compound **7** (90 mg, 44%). MS (ESI): *m*/*z* for [C_18_H_20_N_6_O_4_+H]^+^—calculated, 385.2; found, 385.1. ^1^H nuclear magnetic resonance (NMR) (CDCl_3_): δ 8.62 (1H, d, *J* = 1.8 Hz), 8.22 (1H, brs), 8.14 to 8.10 (2H, m), 7.48 (1H, d, *J* = 8.1 Hz), 2.88 (3H, s), 2.67 (3H, s), and 1.57 (9H, s).

3-Methyl-6-(4-methyl-3-nitrophenyl)-[1,2,4]triazolo[4,3-*b*]pyridazin-8-amine (**8**): Trifluoroacetic acid (2 ml) was added to a solution of carbamate **7** (80 mg, 0.21 mmol) in DCM (10 ml), and the mixture was stirred at room temperature. The reaction was monitored by TLC. Upon completion, the mixture was concentrated under reduced pressure, and the residue was purified by flash column chromatography (DCM/MeOH, 30:1) to yield **8** (64 mg, 100%). MS (ESI): *m*/*z* for [C_13_H_12_N_6_O_2_+H]^+^—calculated, 285.1; found, 285.3. ^1^H NMR [dimethyl sulfoxide (DMSO)–*d*_6_]: δ 8.49 (1H, d, *J* = 1.8 Hz), 8.19 (1H, dd, *J* = 1.9 Hz, 8.0 Hz), 7.68 (1H, d, *J* = 8.1 Hz), 6.65 (1H, s), 2.70 (3H, s), and 2.60 (3H, s).

Ethyl (3-methyl-6-(4-methyl-3-nitrophenyl)-[1,2,4]triazolo[4,3-*b*]pyridazin-8-yl)carbamate (**9**): Triethylamine (0.35 ml, 2.46 mmol) was added to a solution of amine **8** (348 mg, 1.23 mmol) in dry THF (20 ml) at 0°C. After 30 min, ethyl chloroformate (0.34 ml, 2.46 mmol) was added, and the reaction mixture was warmed to room temperature. The reaction was monitored by TLC. Upon completion, water (30 ml) was added, and the aqueous phase was extracted with DCM. The combined organic layers were dried (Na_2_SO_4_), and the solvent was removed under reduced pressure. The crude product was purified by flash column chromatography (EtOAc/petroleum ether, 1:1) to yield **9** (100 mg, 23%). MS (ESI): *m*/*z* for [C_16_H_16_N_6_O_4_+H]^+^—calculated, 357.1; found, 357.1. ^1^H NMR (CDCl_3_): δ 8.60 to 8.59 (2H, m), 8.19 (1H, s), 8.10 (1H, dd, *J* = 1.8 Hz, 8.1 Hz), 7.52 (1H, d, *J* = 8.1 Hz), 4.35 (2H, q, *J* = 7.1 Hz), 2.85 (3H, s), 2.67 (3H, s), and 1.38 (3H, t, *J* = 7.1 Hz).

Ethyl (6-(3-amino-4-methylphenyl)-3-methyl-[1,2,4]triazolo[4,3-*b*]pyridazin-8-yl)carbamate (**10**): SnCl_2_ hydrate (287 mg, 1.28 mmol) was added to a solution of nitrobenzene **9** (91 mg, 0.26 mmol) in EtOH (10 ml). The reaction mixture was heated to reflux and monitored by TLC. Upon completion, the solvent was removed under reduced pressure, and the crude product was purified by flash column chromatography (DCM/MeOH, 50:1) to yield **10** (74 mg, 87%). MS (ESI): *m*/*z* for [C_16_H_18_N_6_O_2_+H]^+^—calculated, 327.1; found, 326.9. ^1^H NMR (CDCl_3_): δ 8.36 (1H, brs), 8.13 (1H, s), 7.32 to 7.30 (2H, m), 7.16 (1H, d, *J* = 8.1 Hz), 4.33 (2H, q, *J* = 7.2 Hz), 3.80 (2H, brs), 2.84 (3H, s), 2.23 (3H, s), and 1.37 (3H, t, *J* = 7.2 Hz).

Ethyl (3-methyl-6-(4-methyl-3-(methylsulfonamido)phenyl)-[1,2,4]triazolo[4,3-*b*]pyridazin-8-yl)carbamate (**11**) (BSP): Methanesulfonyl chloride (2 eq) was added to a solution of amine **10** (1 eq) in DCM (0.037 M), followed by addition of pyridine (0.6 eq). The resulting mixture was stirred at room temperature. The reaction was monitored by TLC. Upon completion, water was added, and the aqueous layer was extracted with DCM. The organic layers were combined and dried (Na_2_SO_4_). The solvent was removed, and the residue was purified by flash column chromatography (DCM/MeOH, 30:1) to give BSP **11** (78%). MS (ESI): *m*/*z* for [C_17_H_20_N_6_O_4_S+H]^+^—calculated, 405.1; found, 405.5. ^1^H NMR (CDCl_3_): δ 8.93 (1H, brs), 8.14 (1H, s), 8.10 (1H, d, *J* = 1.8 Hz), 7.70 (1H, dd, *J* = 1.8 Hz, 7.8 Hz), 7.64 (1H, brs), 7.32 (1H, d, *J* = 8.1 Hz), 4.30 (2H, q, *J* = 7.1 Hz), 3.09 (3H, s), 2.82 (3H, s), 2.44 (3H, s), and 1.34 (3H, t, *J* = 7.2 Hz).

### Cloning, protein expression, and purification

Human BRDs were subcloned into bacteria-expressing vectors [pNIC28-Bsa4 (GenBank EF198106) and pNIC-Bio2 (GenBank JF912191)], expressed, and purified as previously described by Filippakopoulos *et al.* (*1*).

### Thermal stability assay (*T*_m_ shift)

Thermal melting experiments were carried out using an Mx3005P Real-Time PCR machine (Stratagene). Proteins were buffered in 10 mM Hepes buffer (pH 7.5) and 500 mM NaCl and assayed on a 96-well plate at a final concentration of 2 μM in a volume of 20 μl. Compounds were added at a final concentration of 10 or 100 μM. SYPRO Orange Protein Gel Stain (Molecular Probes) was added as a fluorescence probe at a dilution of 1:1000. Excitation and emission filters for the SYPRO Orange dye were set to 465 and 590 nm, respectively. The temperature was raised from 25° to 96°C at a step of 3°C/min, and fluorescence readings were taken at each interval. The temperature dependence of the fluorescence during the protein denaturation process was approximated by the equation

$$y(T)=y_{F}+\frac{y_{U}-y_{F}}{1+e^{\Delta {\text{uG}}_{\left(T\right)}/RT}}$$

where ΔuG is the difference in unfolding free energy between the folded state and the unfolded state, *R* is the gas constant, and *y*_F_ and *y*_U_ are the fluorescence intensities of the probe in the presence of completely folded and unfolded proteins, respectively (*30*). The baselines of the denatured and native states were approximated by a linear fit. The observed temperature shifts (Δ*T*_m_^obs^) were recorded as the difference between the transition midpoints of sample and reference wells containing proteins without ligands in the same plate and were determined by nonlinear least-squares fit. Temperature shifts (Δ*T*_m_^obs^) for three independent measurements per protein/compound are summarized in table S1 and Table 2.

### Biolayer interferometry

Experiments were performed on an Octet RED384 System (FortéBio) at 25°C in 20 mM Hepes (pH 7.5), 150 mM NaCl, and 0.5 mM tris(2-carboxyethyl)phosphine using the FortéBio data acquisition software V.7.1.0.100. Biotinylated BRDs were immobilized onto Super Streptavidin biosensors (Super Streptavidin Dip and Read Biosensors for kinetic no. 18-0011; FortéBio), preequilibrated in the BLI buffer, and quenched in a solution of 5 μM biotin (baseline equilibration for 60 s, peptide loading for 240 s, and quenching for 60 s; shake speed of 1000 rpm at 25°C). The immobilized proteins were subsequently used in association and dissociation measurements performed within a time window of 600 s (baseline equilibration for 60 s, association for 600 s, and dissociation for 600 s; shake speed of 1000 rpm at 25°C). Interference patterns from protein-coated biosensors without proteins were used as controls. After referencing corrections, the subtracted binding interference data were analyzed using the FortéBio data analysis software V.7.1.0.38 (provided with the instrument) following the manufacturer’s protocols.

### Isothermal titration calorimetry

Experiments were carried out on an ITC200 microcalorimeter from MicroCal LLC (GE Healthcare) equipped with a washing module, a reaction cell (volume of 0.2003 ml), and a 40-μl microsyringe. Experiments were carried out in ITC buffer [50 mM Hepes (pH 7.5; 25°C) and 150 mM NaCl] at 15°C while stirring at 1000 rpm. The microsyringe was loaded with a solution of a protein sample (200 to 650 μM in ITC buffer) and was carefully inserted into the calorimetric cell, which was filled with the compound (0.2 ml, 13 to 25 μM in ITC buffer). The system was first allowed to equilibrate until the cell temperature reached 15°C, and an additional delay of 60 s was applied. All titrations were conducted using an initial control injection of 0.3 μl, followed by 38 identical injections of 1 μl for a duration of 2 s (per injection) and with a spacing of 120 s between injections. The titration experiments were designed to ensure complete saturation of the proteins before the final injection. The heats of dilution for the proteins were independent of their concentrations and corresponded to the heats observed from the last injection, after saturation of ligand binding, thus facilitating estimation of the baseline of each titration from the last injection. The collected data were corrected for protein heats of dilution (measured in separate experiments by titrating the proteins into ITC buffer) and deconvoluted using the MicroCal Origin software (supplied with the instrument) to yield enthalpies of binding (Δ*H*) and binding constants (*K*_B_), as previously described in detail by Wiseman *et al.* (*31*). Thermodynamic parameters were calculated using the basic equation of thermodynamics (Δ*G* = Δ*H* − *T*Δ*S* = −*RT*ln*K*_B_, where Δ*G*, Δ*H*, and Δ*S* are the changes in free energy, enthalpy, and entropy of binding, respectively). In all cases, a single binding site model was used (supplied with the MicroCal Origin software package). Dissociation constants and thermodynamic parameters are listed in Table 1.

### Crystallization

Aliquots of the purified proteins were set up for crystallization using a mosquito crystallization robot (TTP Labtech). Coarse screens were typically set up onto Greiner three-well plates using three different drop ratios of precipitant to protein per condition (100 + 50, 75 + 75, and 50 + 100 nl). Initial hits were optimized, further scaling up the drop sizes. All crystallizations were carried out using the sitting-drop vapor diffusion method at 4°C. BRD4(1) crystals with BSP were grown by mixing 200 nl of the protein (9.9 mg/ml and 5 mM final ligand concentration) with 100 nl of reservoir solution containing 0.20 M sodium/potassium tartrate, 0.1 M BT-propane (pH 8.5), 20% polyethylene glycol (PEG) 3350, and 10% ethylene glycol. TAF1L(2) crystals with BSP were grown by mixing 150 nl of the protein (11.2 mg/ml and 10 mM final ligand concentration) with 150 nl of reservoir solution containing 0.1 M MMT [mixture of dl-malic acid and 2-(*N*-morpholino)-ethanesulfonic acid monohydrate] (pH 7.5) and 63% 2-methyl-2,4-pentanediol. BRD9 crystals with BSP were grown by mixing 100 nl of the protein (28 mg/ml and 10 mM final ligand concentration) with 200 nl of reservoir solution containing 0.1 M bis-tris (pH 5.5), 0.2 M NaCl, and 25% PEG3350. Diffraction-quality crystals grew within a few days.

### Data collection and structure solution

BRD4(1) crystals were cryoprotected using the well solution supplemented with additional ethylene glycol and were flash-frozen in liquid nitrogen. Data were collected in-house on a Rigaku FRE rotating anode system equipped with an R-AXIS IV detector at 1.52 Å [BRD4(1)/BSP], at Diamond beamline I02 at a wavelength of 0.9795 Å [TAF1L(2)/BSP], or at Diamond beamline I04 at a wavelength of 0.9795 Å (BRD9/BSP). Indexing and integration were carried out using XDS (*32*, *33*), and scaling was performed with Scala (*34*). Initial phases were calculated by molecular replacement with PHASER (*35*) using the known models of BRD4(1), TAF1L(2), or BRD9 [Protein Data Bank (PDB) accession codes 2OSS, 3HMH, and 3HME, respectively]. Initial models were built by ARP/wARP (*36*) followed by manual building in Coot (*37*). Refinement was carried out in REFMAC5 (*38*). Thermal motions were analyzed using TLS Motion Determination (*39*), and hydrogen atoms were included in late refinement cycles. Data collection and refinement statistics are found in table S4. The model and structure factors have been deposited with PDB accession codes 5IGK [BRD4(1)/BSP], 5IGL [TAF1L(2)/BSP], and 5IGM (BRD9/BSP).

### BSP pull-down assay

BSP pull-downs were performed using two different biotinylated probes (BSP-a and BSP-b) from a lysate of ~2 **×** 10^7^ HEK293T cells per sample. Briefly, to the frozen HEK293T cell pellet, 1.6 ml of ice-cold lysis buffer [50 mM Hepes-NaOH (pH 8.0), 2 mM EDTA, 0.1% NP-40, 10% glycerol, 1 mM phenylmethylsulfonyl fluoride, 1 mM dithiothreitol, and Sigma Protease Inhibitor Cocktail (P8340, 1:500) with 300 mM KCl] was added per 15-cm plate of cells, and the frozen pellet was gently resuspended. Samples were subjected to a freeze/thaw cycle on dry ice until completely frozen (5 to 10 min) and then transferred to a 37°C water bath with agitation until only a small amount of ice remained. Samples were sonicated for 30 s (10 s on–2 s off cycles at an amplitude of 0.35) using a Qsonica 125-W sonicator equipped with a ^1^/_8_-inch probe to shear DNA. Benzonase (1 μl, 250 U/μl; E1014; Sigma-Aldrich) was then added to each sample and incubated at 4°C for 1 hour to further digest chromatin. The resulting samples were centrifuged at 14,000 rpm (20,873*g*) for 20 min at 4°C, and the supernatant was transferred to fresh 2-ml tubes. Biotinylated BSP probes [50 nmol conjugated to 20 μl of MyOne Streptavidin C1 Dynabeads (65002; Invitrogen) for at least an hour in 1× phosphate-buffered saline (PBS)] were washed with lysis buffer, and an equal bead volume was subsequently aliquoted between centrifuged cell lysates. The mixture was incubated for 2 hours at 4°C with gentle agitation (nutator) with or without competition from 30 nmol of BSP. Beads were then pelleted by centrifugation (1000 rpm for 5 s), and tubes were placed on a cold magnetic rack (on ice) to collect the beads on the side of the tubes. The supernatant was removed slowly with a pipette, and the beads were washed once with 1 ml of cold lysis buffer with 300 mM KCl and washed twice with lysis buffer containing 100 mM KCl. The beads were then transferred to a fresh 1.7-ml tube with 1 ml of 20 mM tris-HCl (pH 8.0) and 2 mM CaCl_2_. After the last wash, the samples were quickly centrifuged, and the last drops of liquid were removed with a fine pipette.

Binding of BSP to CECR2 was evaluated in Flp-In T-REx HEK293 cells stably expressing 3×FLAG CECR2 (accession no. BC166664) or an empty 3×FLAG control (using lysis buffer containing 300 mM KCl). After pull-down, proteins were eluted off the beads by adding 40 μl of 2× Laemmli buffer and heating the samples to 65°C for 15 min. The samples were then cooled to room temperature, quickly centrifuged, and placed on a magnetic rack to collect the beads on the side of the tubes. The supernatants were then transferred to fresh tubes and stored at −40°C until Western blots were performed. One percent of input and 25% of purified proteins were separated by SDS–polyacrylamide gel electrophoresis and transferred onto nitrocellulose membranes. The membranes were blocked in tris-buffered saline containing nonfat milk (5 mg/ml) and 1% Tween 20 for 1 hour at room temperature. Blots were probed for FLAG (1:5000; F1804; Sigma-Aldrich) or β-tubulin (1:5000; E7; Developmental Studies Hybridoma Bank) at the University of Iowa. Detection on film was performed by chemiluminescence using the LumiGLO reagent (1:20; 7003; Cell Signaling Technology).

### Trypsin digestion of affinity-purified proteins

After pull-down on magnetic beads, samples were resuspended in 7.5 μl of 20 mM tris-HCl (pH 8.0) containing 500 ng of trypsin (Trypsin Singles, T7575; Sigma-Aldrich), and the suspension was incubated at 37°C with agitation overnight on an angled rotating wheel (~15 hours). After this first incubation, samples were quickly centrifuged and then magnetized, and the supernatants were transferred to a fresh tube. Another 250 ng of trypsin was added [in 2.5 μl of 20 mM tris-HCl (pH 8.0)], and the resulting sample was incubated at 37°C for 3 to 4 hours without agitation. Formic acid was then added to a final concentration of 2% (from 50% stock solution) and stored at −80°C.

### MS analysis

Pull-down samples and controls were analyzed by MS, as previously described by Barsyte-Lovejoy *et al.* (*40*). Briefly, 5 μl of each sample (representing ~50% of the sample) was directly loaded at a flow rate of 400 nl/min onto a 75 μm **×** 12 cm emitter packed with a 3-μm ReproSil-Pur C18-AQ (Dr. Maisch GmbH HPLC). The peptides were eluted from the column over a 90-min gradient generated by a NanoLC-Ultra 1D Plus nanopump (Eksigent) and analyzed on a TripleTOF 5600 Instrument (AB SCIEX). The gradient was delivered at a flow rate of 200 nl/min starting from 2% acetonitrile with 0.1% formic acid, ramping up to 35% acetonitrile with 0.1% formic acid over 90 min, followed by a 15-min cleanup at 80% acetonitrile with 0.1% formic acid and a 15-min equilibration period back to 2% acetonitrile with 0.1% formic acid, for a total of 120 min. To minimize carryover between each sample, we washed the analytical column for 3 hours by running an alternating sawtooth gradient from 35% acetonitrile with 0.1% formic acid to 80% acetonitrile with 0.1% formic acid, holding each gradient concentration for 5 min. Analytical column and instrument performance were verified after each sample by loading 30 fmol of bovine serum albumin (BSA) tryptic peptide standard (Michrom Bioresources Inc.) with 60 fmol of α-casein tryptic digest and by running a short 30-min gradient. Time-of-flight (TOF) MS calibration was performed on BSA reference ions before the next sample was run to adjust for mass drift and to verify peak intensity. The instrument method was set to discovery or information-dependent acquisition mode, which consisted of one 250-ms TOF MS1 survey scan from 400 to 1300 Da, followed by twenty 100-ms MS2 candidate ion scans from 100 to 2000 Da in high-sensitivity mode. Only ions with charges of 2+ to 4+, which exceeded a threshold of 200 cps, were selected for MS2, and former precursors were excluded for 10 s after one occurrence.

### MS data analysis

MS data generated by TripleTOF 5600 were stored, searched, and analyzed using the ProHits laboratory information management system platform (*41*). Within ProHits, the resulting WIFF files were first converted into an MGF format using the WIFF2MGF converter and into an mzML format using ProteoWizard (v3.0.4468) and the AB SCIEX MS Data Converter (V1.3 beta) and then searched using Mascot (v2.3.02) and Comet (v2012.02 rev.0). The spectra were searched with the RefSeq database (version 53; 28 May 2014) acquired from the National Center for Biotechnology Information (NCBI) against a total of 34,374 human and adenovirus sequences supplemented with “common contaminants” from the Max Planck Institute (http://141.61.102.106:8080/share.cgi?ssid=0f2gfuB) and the Global Proteome Machine (www.thegpm.org/crap/index.html). The database parameters were set to search for tryptic cleavages, allowing up to two missed cleavage sites per peptide, with a mass tolerance of 40 parts per million for precursors with charges of 2+ to 4+ and a tolerance of ±0.15 atomic mass units for fragment ions. Variable modifications were selected for deamidated asparagine and glutamine and for oxidized methionine. The results from each search engine were analyzed through TPP [the Trans-Proteomic Pipeline (*42*) v4.6 OCCUPY rev 3] by means of the iProphet pipeline (*43*). The resulting MS data were presented in a bar graph displaying the ratio of spectral counts obtained for every BRD-containing protein in the presence and absence of competing nonbiotinylated BSP. All MS files used in this study were also deposited at MassIVE (http://massive.ucsd.edu) under MassIVE ID MSV000079365.

### Cell culture

Human cell lines (K562, KASUMI-1, and MV4;11) (*44*–*46*) were obtained from the American Type Culture Collection and the Leibnitz Institute Deutsche Sammlung von Mikroorganismen und Zellkulturen (German Collection of Microorganisms and Cell Cultures) (www.dsmz.de). Cell lines were cultured in RPMI 1640 medium (catalog no. 61870-044; Gibco) containing 10% fetal calf serum (catalog no. 2-01F10-I; BioConcept), penicillin (100 U/ml), and streptomycin (100 U/ml) (catalog no. 15140-122; Gibco). The OCI-AML3 cell line (*47*) was maintained in α minimum essential medium (catalog no. BE12-169F; BioWhittaker) supplemented with 20% heat-inactivated fetal calf serum (no. A15-152; PAA). HEK293T cells were grown in Dulbecco’s modified Eagle’s medium supplemented with 5% fetal bovine serum (catalog no. 12483-020; Gibco), 5% cosmic calf serum (catalog no. SH30087.03; HyClone), and penicillin-streptomycin (catalog no. 30-002-CI; Corning). Cells were grown at 37°C in a humidified cabinet under 5% CO_2_ (Heraeus Function Line).

### In vitro cytotoxicity assays

Cytotoxic activity of BSP on leukemic cell lines was assessed using two different colorimetric assays. Cell viability was assessed using Trypan blue (Sigma). Cells were harvested from exponential phase cultures and plated on 96-well opaque flat-bottom plates at a cell density of 4 **×** 10^4^ cells per well (50 μl). After 2 to 4 hours of recovery, 50 μl of a medium containing DMSO (vehicle) or the test compound was added to the wells. For each concentration, cells were plated in quadruplicate. Cells were exposed to the compound for 48 and 72 hours before 10 μl of WST-1 reagent (catalog no. 05015944001; Roche) was added to every well. After 30 s on an orbital shaker and further incubation for 2 hours, absorbance of the samples was measured with an enzyme-linked immunosorbent assay (ELISA) plate reader (Synergy H1 Hybrid Multi-Mode Microplate Reader) at a wavelength of 450 versus 650 nm (background). Samples were blanked with a control well, and the percentage of surviving cells were compared to controls (fig. S3). Cytotoxic activity of JQ1 and BSP on leukemic cell lines was also assessed using the colorimetric CellTiter Aqueous Non-Radioactive Cell Proliferation Assay (Promega). Cell viability was assessed using Trypan blue (Sigma). Cells were harvested from exponential phase cultures and plated on 96-well opaque flat-bottom plates at a cell density of 2 **×** 10^5^ cells per well (100 μl). After 2 to 4 hours of recovery, 100 μl of a medium containing DMSO (vehicle) or the test compound was added to the wells. For each concentration, cells were plated in triplicate. Cells were exposed to the compound for 72 hours before the addition of 40 μl of 3-(4,5-dimethylthiazol-2-yl)-5-(3-carboxymethoxyphenyl)-2-(4-sulfophenyl)-2H-tetrazolium (MTS), in the presence of phenazine methosulfate (PMS) to each well. After 30 s on an orbital shaker and further incubation for 2 hours, absorbance of the samples at 485 nm was measured with an ELISA plate reader (PHERAstar; BMG LABTECH) (fig. S4A).

### NCI-60 growth inhibition determination

BSP was submitted to the NCI Human Tumor Cell Line Screen (https://dtp.cancer.gov/discovery_development/nci-60/) and profiled against 60 tumor cell lines, first at a single dose of 10 μM and then in a serial dilution of five concentrations, following the NCI standard screening protocol (*48*).

### Binding against human recombinant ligands and ion receptors (CEREP)

Selectivity profiling (ExpresSProfile) was performed on BSP against 104 ligand receptors, ion channels, and transport proteins by CEREP using the manufacturer’s protocols. Data were analyzed in Microsoft Excel (Microsoft Corp.).

### Clonogenic and replating assays in methylcellulose

The impact of BSP on the clonogenic potential of cells was assessed in methylcellulose cultures at different concentrations (0.1, 0.5, and 1.0 μM) or in DMSO (vehicle control). KASUMI-1 and MV4;11 cells were plated in methylcellulose supplemented with human cytokines (MethoCult H4535; STEMCELL Technologies), whereas K562 and MV4;11 cells were plated in methylcellulose without additional cytokines (5510; StemAlpha) at 2 **×** 10^3^ cells per plate. All plates were incubated at 37°C and 5% CO_2_ for 6 to 10 days before the number of colonies was counted and before viable cells were harvested. Cytospots were prepared by centrifuging 10^5^ cells at 300 rpm for 3 min using a Shandon Cytospin 3 centrifuge. Cytospots were stained with Wright-Giemsa stain and analyzed with an Olympus BX62 or Nikon TI microscope at ×60 magnification.

### Cell cycle analysis

Cells were treated in liquid culture with increasing concentrations of BSP or JQ1. After 48 hours, cells were washed twice with PBS, fixed with ice-cold 70% ethanol, and stained in a solution containing propidium iodide (50 μg/ml; P4684; Sigma), ribonuclease (10 μg/ml; 10109142001; Roche), and 1% Triton X-100 in PBS. DNA content was measured on an Accuri C6 cytometer (BD Biosciences), and data were analyzed using the FlowJo software suite (TreeStar Inc.). Experiments were repeated three times.

### Inhibitor combination analysis

Cell lines (MV4;11 and K562 from the log growth phase in RPMI–10% fetal calf serum) were treated with inhibitors (JQ1 or BSP) individually or in combination [JQ1: 0.7 to 11,392 nM (15× concentration); BSP: 37 to 4728 nM (8× concentration)]. Cell survival was determined using a WST-1 assay (no. 05015944001; Roche). Raw data were blanked and normalized to DMSO-treated controls. Data sets were log-transformed and normalized individually, and the combination index (*49*) was calculated for each inhibitor combination using the CompuSyn software suite (ComboSyn Inc.).

### Fluorescence recovery after photobleaching

FRAP studies were performed using a protocol previously described by Philpott *et al.* (*50*). In brief, U2OS cells were transfected (Lipofectamine; Invitrogen) with mammalian overexpression constructs encoding GFP chimeras with BRD4 or BRD9. The FRAP and imaging system consisted of a Zeiss LSM 710 scanhead (Zeiss GmbH) coupled to an inverted Zeiss Axio Observer.Z1 microscope equipped with a high–numerical aperture (1.3) ×40 oil immersion objective (Zeiss GmbH) equipped with a heated chamber set to 37°C. FRAP and GFP fluorescence imaging were carried out with an argon-ion laser (488 nm) and with a piezomultiplier tube detector set to detect fluorescence between 500 and 550 nm. A 5-μm^2^ region of the nucleus was selected, and the region was bleached after five prescans. A time-lapse series was then taken to record GFP recovery using 1% of the power used for bleaching at an interval of 0.25 s. The image data sets and fluorescence recovery data were exported from the ZEN 2010 microscope control software into Origin v.7. The average intensity at each imaging time point was measured for three regions of interest: the bleached region (*I*_*t*_), the total cell nucleus (*T*_*t*_), and a random region outside the cell for background subtraction (BG). The relative fluorescence signal in the bleached region was calculated for each time point *t*, with the following formula (*51*)

$$\left(T_{\text{average prebleach}}-\text{BG})(I_{t}-\text{BG})/(T_{t}-\text{BG})(I_{\text{average prebleach}}-\text{BG}\right)$$

The baseline was normalized to zero, and the prebleach was normalized to 1. Half times of recovery were calculated from the individual curves and presented as the mean. Paired *t* tests were used to generate *P* values for comparisons between two groups.

### RNA extraction

Cells were seeded at 2 **×** 10^5^ cells/ml on the day before treatment. Treatments were performed so that a final concentration of 0.1% DMSO (catalog no. D1435; Sigma) was achieved, and cells were incubated with the vehicle or test compound for 6 hours before isolation of RNA. Total RNA was isolated using a standard TRIzol (Invitrogen) protocol and prepared using RNeasy columns (catalog no. 74106 plus; Qiagen). RNA was quantified using a NanoDrop spectrophotometer (model ND1000; Thermo Fisher Scientific), and integrity was assessed on a BioAnalyzer (2100; Agilent Laboratories). All samples had an RNA integrity number of ≥9.

### Genome-wide expression analysis

mRNA samples were processed using the Illumina TotalPrep-96 RNA Amplification Kit followed by the Illumina Whole-Genome Gene Expression Direct Hybridization Assay. The labeled complementary RNA was then hybridized on Illumina HumanHT-12 v4 bead chips (Illumina Inc.). Chips were processed on an Illumina iScan Scanner, and the Illumina GenomeStudio (v.1.9.0; Illumina Inc.) was used to generate bead files. GenomeStudio data were processed in R (v.3.2) (*52*) using Bioconductor (v.3.1) (*53*) and the lumi package (v.2.20.2) (*54*). Quality controls were carried out using the arrayQualityMetrics package (v.3.24.0) (*55*), taking into account array intensity distributions, distance between arrays, and variance mean dependence. Principal components analysis was used to decide which arrays to process together. Background correction followed by variance-stabilizing transform (*56*) and quantiles between microarrays normalization were carried out with the lumi package. From the 47,231 probe sets available on the HumanHT12 V4 chip, removal of unexpressed probes resulted in 24,283 probe sets. A linear model was applied using the limma package (v.3.24.13) (*57*), followed by empirical Bayesian analysis, to determine differential expression between untreated and treated samples. Genes were considered to be differentially expressed if the adjusted *P* value [calculated using the Benjamini-Hochberg method (*58*) to minimize false discovery rate (FDR)] was less than 0.05 and the mean level of expression was greater than 1.5-fold. Gene Ontology (GO) enrichment analysis was performed with the topGO package (v.2.20.0) (*59*) using the weight01 algorithm and Fisher’s exact test to calculate the significance of a GO term. A cutoff value of 0.01 was imposed on the adjusted *P* values to report enriched terms. Genes exhibiting a differential expression upon BSP or JQ1 treatment (Benjamini-Hochberg adjusted *P* < 0.01) were further subjected to enrichment analyses in the MetaCore software suite (MetaCore v.6.19.65960; Thomson Reuters) to reveal signaling and metabolic pathways, as well as cell process networks overrepresented in the differentially expressed gene sets. *P* values for pathway enrichment analysis were calculated using the formula for hypergeometric distribution, reflecting the probability for a pathway to arise by chance. Statistically enriched pathways and networks were identified using a threshold FDR of 0.001. Gene expression data have been deposited in NCBI’s Gene Expression Omnibus and are accessible through GEO Series accession number GSE78830.

### Gene set analysis and GSEA

Gene expression data were further filtered to remove unannotated genes, resulting in 18,754 probes. Multiprobe profiles were averaged using the collapseRows R function (*60*), resulting in 13,620 unique genes, which were imported into the Broad GSEA suite (v.2.2.0) (*61*) as a collapsed set. Gene set analysis was performed with the piano package (v.1.8.2) (*62*) using the MaxMean method (*63*), with 1000 permutations and with minimum and maximum gene sets of 15 and 500, respectively, against the 50 hallmark (h) gene sets from the MSigDB (v.5.0). Resulting gene sets with a nominal *P* value of 0.05 were considered significant. Distinct nondirectional and directional network maps were visualized with the piano package.

GSEA was performed with the Broad GSEA suite (v.2.2.0) (*61*) in a collection of 4725 curated gene sets (c2), 615 transcription factors (c3), and 50 hallmarks (h) from MSigDB (v.5.0). Gene sets with less than 15 genes or more than 500 genes were excluded from the analysis, whereas gene sets with an FDR of ≤0.25 and a nominal *P* value of ≤0.05 were considered significant. Gene ranking was performed with the weighted enrichment score using a two-sided signal-to-noise ratio, and *P* values were calculated using 1000 permutations of the gene set.

### JQ1 gene signatures

The AML JQ1 signature was constructed using the gene expression data (GEO data set: GSE29799) after a 24-hour treatment of THP1 cells with 250 nM JQ1, as reported by Zuber *et al.* (*6*). Differentially expressed genes with an adjusted *P* value of <0.001 and a log fold change of >2, which were down-regulated (185 genes), were used as a ranked list to construct a gene set for subsequent GSEA. A smaller JQ1 signature consisting of 36 genes previously reported by Puissant *et al.* (*27*) to be down-regulated by JQ1 in neuroblastoma, multiple myeloma, and AML was also used to construct a gene set for subsequent GSEA.

### Quantitative real-time polymerase chain reaction

One microgram of RNA was used to prepare cDNA using the iScript cDNA synthesis kit (catalog no. 1708891; Bio-Rad) according to the manufacturer’s instructions. The resulting cDNA was diluted 1:10 before qRT-PCR was performed. Samples were prepared on 384-well plates with a final reaction volume of 10 μl containing SYBR Select Master Mix (catalog no. 4472908; Thermo Fisher Scientific), 0.4 μM forward and reverse primers, and 2 μl of diluted cDNA. All reactions were run on a QuantStudio 6 Flex Real-Time PCR system (Thermo Fisher Scientific) under the following conditions: 1 cycle at 50°C for 2 min and then 1 cycle at 95°C for 2 min, followed by 40 cycles at 95°C for 15 s and then 60°C for 1 min each. Data were analyzed using the 2^−ΔΔ*C*T^ method (*64*), and sample normalization was performed using 18*S* ribosomal RNA as the endogenous control and *SDHA* as the reference gene. Values are presented as means ± SD from three biological replicates. *P* values are presented such that ****P < 0.001, ***P < 0.005, **P < 0.01, and *P < 0.05 and were evaluated with one-step ANOVA followed by Dunnett’s test (performed in GraphPad Prism v.6). Primers are listed in table S5.

## Supplementary Material

http://advances.sciencemag.org/cgi/content/full/2/10/e1600760/DC1

## SUPPLEMENTARY MATERIALS

Supplementary material for this article is available at http://advances.sciencemag.org/cgi/content/full/2/10/e1600760/DC1 fig. S1. Topology of BRD cavity and binding of chemical scaffolds containing different potential expansion vectors. fig. S2. Structure-activity relationship of the triazolopyridazine class leading to BSP. fig. S3. BSP inhibits growth of cancer cell lines. fig. S4. Effect of BSP and JQ1 on leukemia cell lines. fig. S5. Effect of BSP and JQ1 on leukemia cell lines. fig. S6. Gene expression GO enrichment (biological processes). fig. S7. Gene expression after inhibition of leukemia cell lines with BSP or JQ1. fig. S8. GSEA of K562 and KASUMI-1 cell lines after BSP treatment. fig. S9. GSEA of MV4;11 and OCI-AML3 cell lines after BSP treatment. fig. S10. Effect of BSP on BET-specific genes. fig. S11. Expression of BRD-containing proteins in leukemic cell lines. fig. S12. Effects of the selective inhibition of different BRD subfamilies on transcriptional programs in leukemias. fig. S13. Transcriptional response in leukemia cell lines and inhibitor combination. fig. S14. GSEA comparison of BSP and JQ1 effects on leukemias. fig. S15. BSP profile of cellular receptor activity (ExpresSProfile; CEREP). table S1. Differential scanning fluorimetry profiling of triazolopyridazines against a panel of BRD modules. table S2. MetaCore analysis of gene expression data. table S3. BSP profile of cellular receptor activity data (ExpresSProfile; CEREP). table S4. Data collection and refinement statistics for BRD-BSP complexes. table S5. Primers used for qRT-PCR.
