# Supplementary material for: Promiscuous targeting of bromodomains by bromosporine identifies BET proteins as master regulators of primary transcription response in leukemia
Source: Sci Adv. 2016 Oct 12;2(10):e1600760. doi: 10.1126/sciadv.1600760 (PMC5061470; doi:10.1126/sciadv.1600760)
Supplement: http://advances.sciencemag.org/cgi/content/full/2/10/e1600760/DC1 [file supp_2_10_e1600760__index.html]

Science Advances | Science Advances

## Supplementary Materials

**This PDF file includes:**

- fig. S1. Topology of BRD cavity and binding of chemical scaffolds containing different potential expansion vectors.
- fig. S2. Structure-activity relationship of the triazolopyridazine class leading to BSP.
- fig. S3. BSP inhibits growth of cancer cell lines.
- fig. S4. Effect of BSP and JQ1 on leukemia cell lines.
- fig. S5. Effect of BSP and JQ1 on leukemia cell lines.
- fig. S6. Gene expression GO enrichment (biological processes).
- fig. S7. Gene expression after inhibition of leukemia cell lines with BSP or JQ1.
- fig. S8. GSEA of K562 and KASUMI-1 cell lines after BSP treatment.
- fig. S9. GSEA of MV4;11 and OCI-AML3 cell lines after BSP treatment.
- fig. S10. Effect of BSP on BET-specific genes.
- fig. S11. Expression of BRD-containing proteins in leukemic cell lines.
- fig. S12. Effects of the selective inhibition of different BRD subfamilies on transcriptional programs in leukemias.
- fig. S13. Transcriptional response in leukemia cell lines and inhibitor combination.
- fig. S14. GSEA comparison of BSP and JQ1 effects on leukemias.
- fig. S15. BSP profile of cellular receptor activity (ExpresSProfile; CEREP).
- Legends for tables S1 and S2
- table S3. BSP profile of cellular receptor activity data (ExpresSProfile; CEREP).
- table S4. Data collection and refinement statistics for BRD-BSP complexes.
- table S5. Primers used for qRT-PCR.

Download PDF

**Other Supplementary Material for this manuscript includes the following:**

- table S1 (Microsoft Excel format). Differential scanning fluorimetry profiling of triazolopyridazines against a panel of BRD modules.
- table S2 (Microsoft Excel format). MetaCore analysis of gene expression data.

Download tables S1 and S2

**Files in this Data Supplement:**

- Adobe PDF - 1600760\_SM.pdf
