# Supplementary material for: Promiscuous targeting of bromodomains by bromosporine identifies BET proteins as master regulators of primary transcription response in leukemia
Source: Sci Adv. 2016 Oct 12;2(10):e1600760. doi: 10.1126/sciadv.1600760 (PMC5061470; doi:10.1126/sciadv.1600760)
Supplement: http://advances.sciencemag.org/cgi/content/full/2/10/e1600760/DC1 [file 1600760_SM.pdf]

Science Advances
AAAS
advances.sciencemag.org/cgi/content/full/2/10/e1600760/DC1

Supplementary Materials for

Promiscuous targeting of bromodomains by bromosporine identifies BET proteins as master regulators of primary transcription response in leukemia

Sarah Picaud, Katharina Leonards, Jean-Philippe Lambert, Oliver Dovey, Christopher Wells, Oleg Fedorov, Octovia Monteiro, Takao Fujisawa, Chen-Yi Wang, Hannah Lingard, Cynthia Tallant, Nikzad Nikbin, Lucie Guetzoyan, Richard Ingham, Steven V. Ley, Paul Brennan, Susanne Muller, Anastasia Samsonova, Anne-Claude Gingras, Juerg Schwaller, George Vassiliou, Stefan Knapp, Panagis Filippakopoulos

Published 12 October 2016, *Sci. Adv.* **2**, e1600760 (2016)
DOI: 10.1126/sciadv.1600760

The PDF file includes:

- fig. S1. Topology of BRD cavity and binding of chemical scaffolds containing different potential expansion vectors.
- fig. S2. Structure-activity relationship of the triazolopyridazine class leading to BSP.
- fig. S3. BSP inhibits growth of cancer cell lines.
- fig. S4. Effect of BSP and JQ1 on leukemia cell lines.
- fig. S5. Effect of BSP and JQ1 on leukemia cell lines.
- fig. S6. Gene expression GO enrichment (biological processes).
- fig. S7. Gene expression after inhibition of leukemia cell lines with BSP or JQ1.
- fig. S8. GSEA of K562 and KASUMI-1 cell lines after BSP treatment.
- fig. S9. GSEA of MV4;11 and OCI-AML3 cell lines after BSP treatment.
- fig. S10. Effect of BSP on BET-specific genes.
- fig. S11. Expression of BRD-containing proteins in leukemic cell lines.
- fig. S12. Effects of the selective inhibition of different BRD subfamilies on transcriptional programs in leukemias.
- fig. S13. Transcriptional response in leukemia cell lines and inhibitor combination.
- fig. S14. GSEA comparison of BSP and JQ1 effects on leukemias.
- fig. S15. BSP profile of cellular receptor activity (ExpresSProfile; CEREP).
- Legends for tables S1 and S2

- table S3. BSP profile of cellular receptor activity data (ExpresSProfile; CEREP).
- table S4. Data collection and refinement statistics for BRD-BSP complexes.
- table S5. Primers used for qRT-PCR.

Other Supplementary Material for this manuscript includes the following:
(available at advances.sciencemag.org/cgi/content/full/2/10/e1600760/DC1)

- table S1 (Microsoft Excel format). Differential scanning fluorimetry profiling of triazolopyridazines against a panel of BRD modules.
- table S2 (Microsoft Excel format). MetaCore analysis of gene expression data.

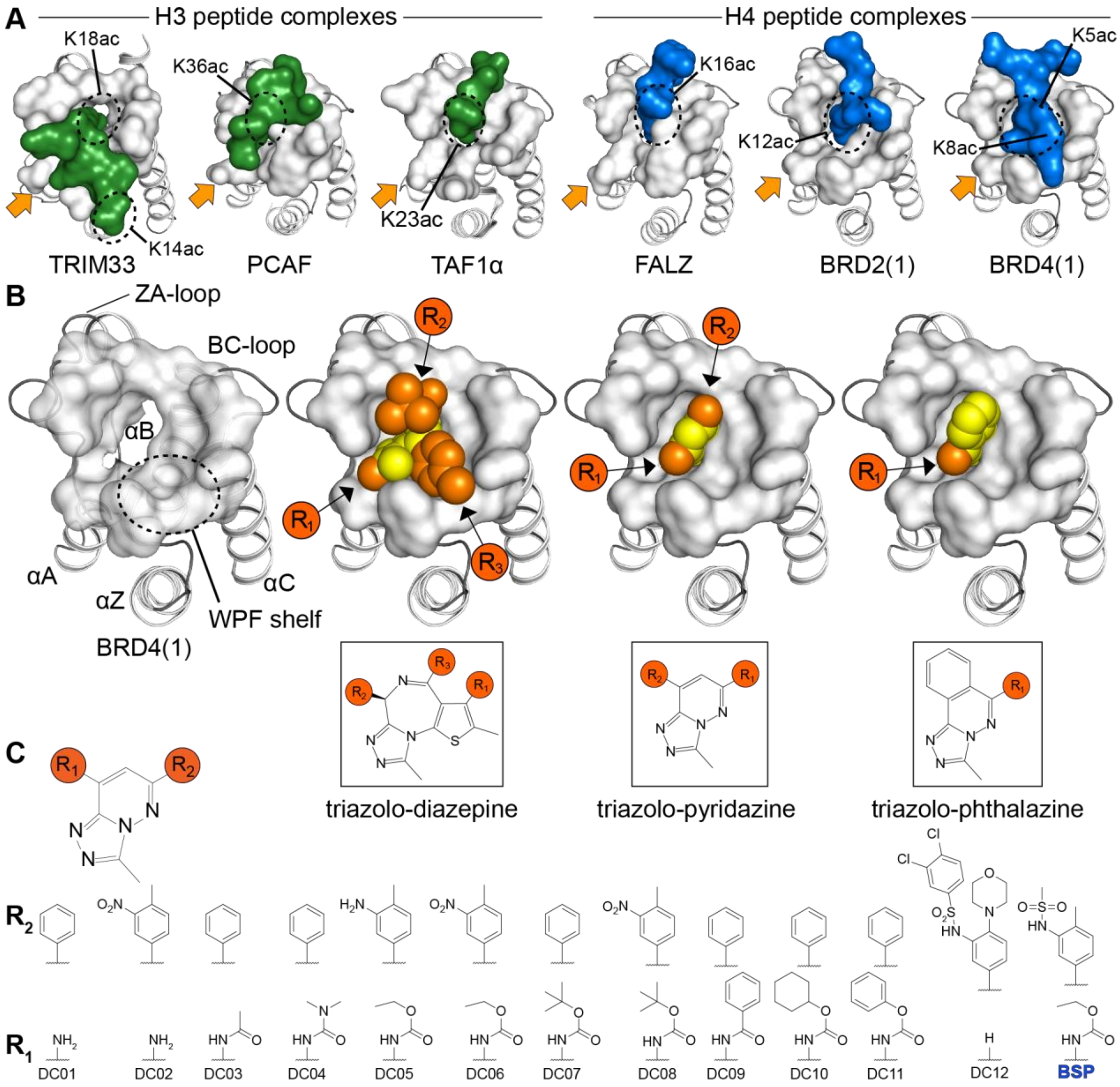

fig. S1. Topology of BRD cavity and binding of chemical scaffolds containing different potential expansion vectors. (**A**) Histone H3 (left, TRIM33/H3K9me3K14ac18ac, PDB ID: 3U5O; PCAF/H3K36ac, PDB ID: 2RNX (NMR); TAF1α/H3K23ac, PDB ID: 3O34) and H4 (right, FALZ/H4K12ac, PDB ID: 3QZS; BRD2(1)/H4K12ac, PDB ID: 2DVQ; BRD4(1)/K5acK8ac, PDB ID: 3UVW) peptide complexes highlight the overlay of the peptide path on top of the bromodomain acetyl-lysine recognition cavity. Proteins are illustrated in cartoon representation with the acetyl-lysine binding sites shown as surfaces. Peptides are rendered as solid volumes highlighting the direction of binding within the BRD cavities. The arrow highlights a channel common to all BRD structures formed by the ZA-loop and helix A. (**B**) Crystal structures of BRD4(1) complexes with small molecule scaffolds (shown as CPK models) containing different functional groups (shown in orange) allowing for expansion that can best mimic peptide binding. Available scaffolds provide three expansion vectors in the case of triazolo-diazepines, two in the case of triazolo-pyridazines and one in the case of triazolo-

phthalazines (R_1-R_3 highlighted in orange). (**C**) Core triazolo-pyridazine scaffold and R substituents used to establish BSP. Functional groups (R_1 and R_2) are shown for each compound within the class

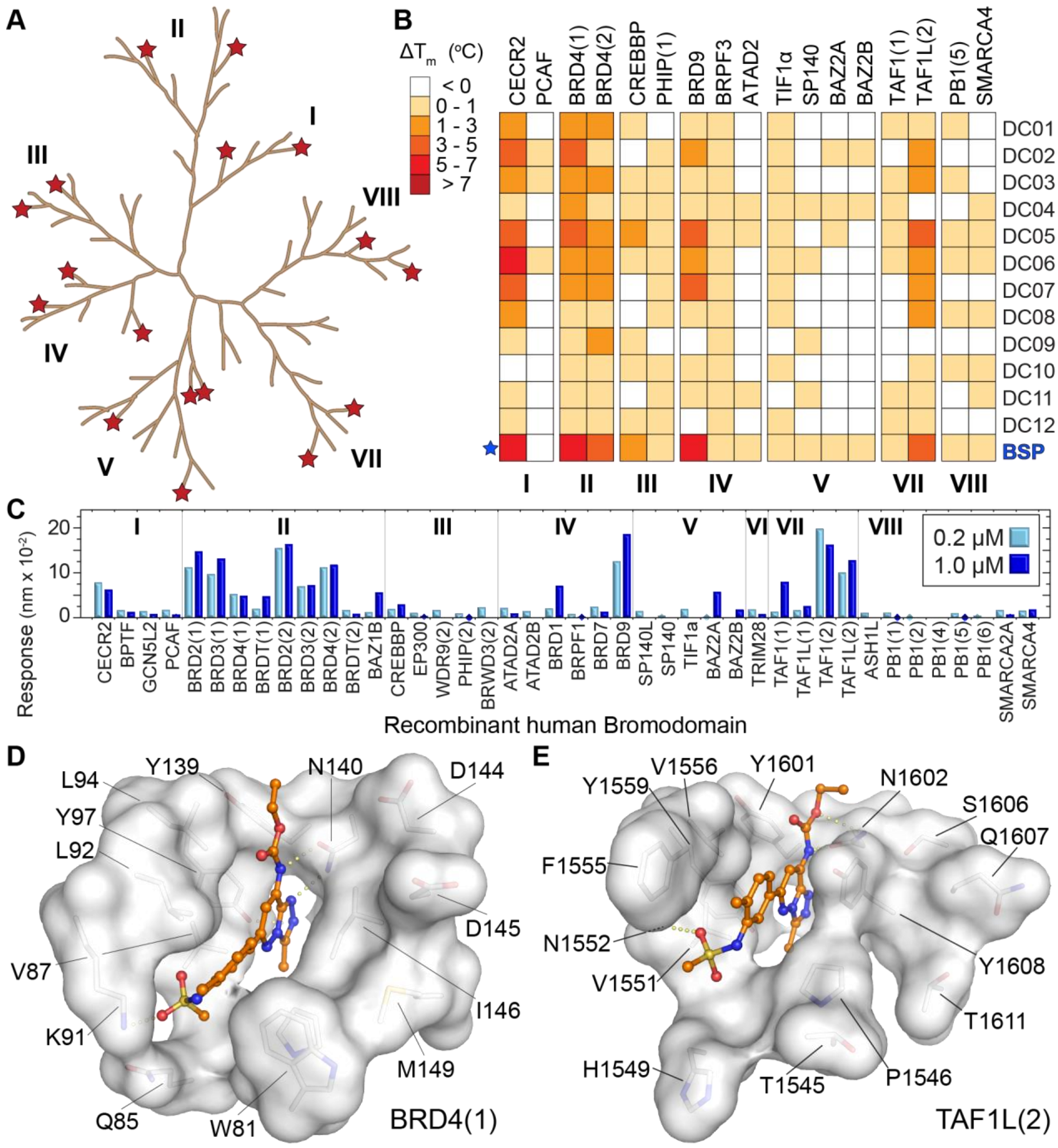

fig. S2. Structure-activity relationship of the triazolopyridazine class leading to BSP. (**A**/**B**) Family-wide SAR. Bromodomains representing each structural sub-class of the human bromodomain family are annotated with a red star on the phylogenetic tree (shown on the left), were screened against a focused series of triazolopyridazine-based compounds employing differential scanning fluorimetry. The heatmap on the right represents thermal melt shifts (ΔT_m) for each protein and is coloured as indicated in the inset, highlighting the promiscuous character of the compounds. BSP (highlighted in blue) was profiled further and is referred to as BSP given its promiscuous character. (**C**) Biolayer Interferometry (BLI) profiling of BSP against the family of human bromodomains. Recombinant biotinylated human bromodomains representing all sub-

families were immobilized on biosensors and binding to 0.2 or 1.0 μM of BSP was assessed. The compound binds to most classes of human bromodomains. (**D**) Crystal structure of the first bromodomain of human BRD4 in complex with BSP. The acetyl-lysine recognition cavity of the BRD module is shown as a surface with key residues highlighted. The reverse sulphonamide function of BSP (R_1 substituent of the triazolopyridazine scaffold) extends towards the front opening of the pocket initiating backbone interactions with the ZA-loop and packing under K91. The ethyl-carbamate function of BSP (R_2 substituent of the triazolopyridazine scaffold) initiates an interaction to the conserved asparagine and extends on the back of the pocket. (**E**) Crystal structure of the second bromodomain of human TAF1L in complex with BSP. As in (**D**) the compound engages the protein through hydrogen bonding to the conserved asparagine (N1602), while rotation of the reverse sulphonamide allows reposition of this functional group under F1555, occupying the left portion of the ZA-loop while initiating backbone interactions with N1552. The compound is shown in ball and stick representation in (**D**) and (**E**).

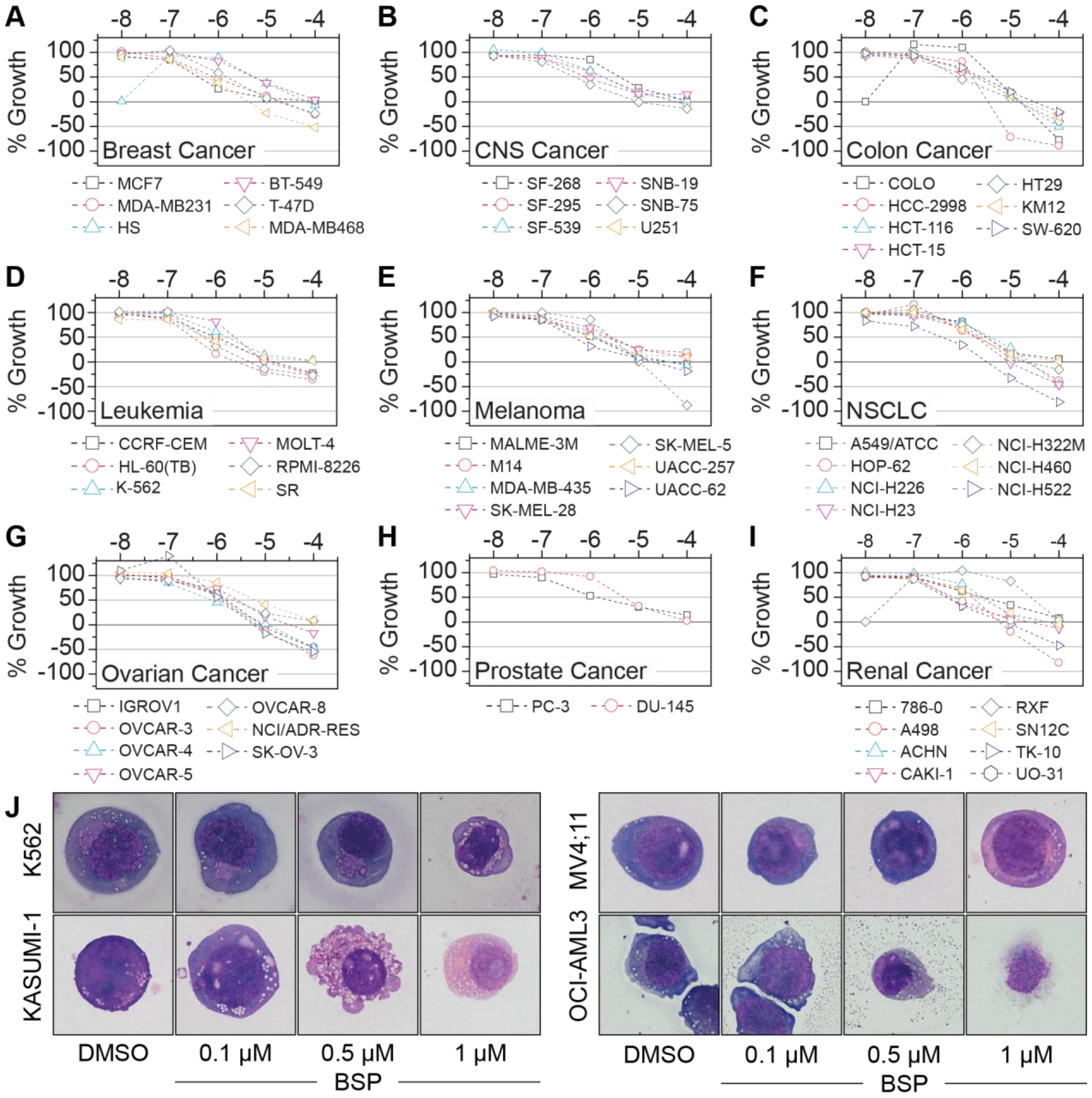

fig. S3. BSP inhibits growth of cancer cell lines. (**A-I**) Growth inhibition curves were generated by treating the National Cancer Institute's (NCI) panel of cell lines (NCI-60) for 48 hours with a serial dilution of BSP. The compound potently inhibits growth in most tumour backgrounds. (**J**) K562, KASUMI-1, MV4;11 and OCI-AML3 cells were treated for 48 hours with 0.1, 0.5 or 1 μM of BSP. Images are shown at x60 magnification.

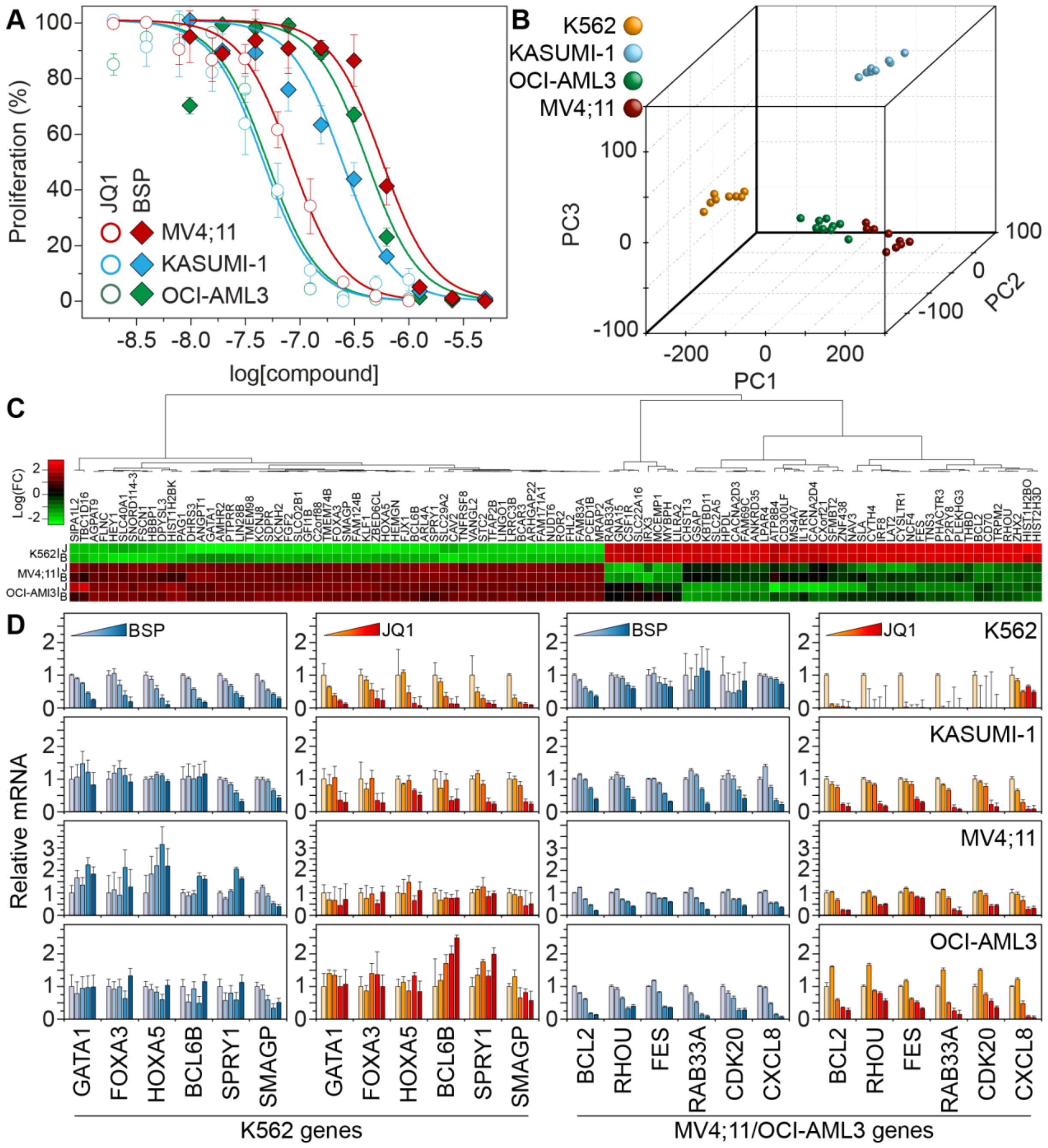

fig. S4. Effect of BSP and JQ1 on leukemia cell lines. (**A**) Cell proliferation assay in MV4;11, KASUMI-1 and OCI-AML3 cell lines using JQ1 and BSP. No significant effect was observed in K562 cells with either inhibitor in the concentration range tested. IC50 values for JQ1 (MV4;11: 0.0802 μM, KASUMI-1: 0.0427 μM and OCI-AML3: 0.0495 μM) and BSP (MV4;11: 0.5793 μM, KASUMI-1: 0.2067 μM and OCI-AML3: 0.3990 μM) were calculated for the three cell lines. (**B**) Principal Component Analysis of gene expression data from Illumina HumanHT-12 v4 beadchip micro arrays performed in the four leukemic cell lines. Samples clustered together by cell line and treatment without any significant outliers. (**C**) Genes that exhibited 10-fold difference in their differential expression between K562 or MV4;11/OCI-AML3 treatments with

JQ1 or BSP (annotated as ‘J’ and ‘B’ respectively). The heatmap represents fold changes as indicated in the inset. (**D**) Quantitative Real Time PCR validation of genes sensitive to K562 BSP or JQ1 inhibition (left) or MV4;11/OCI-AML3 inhibition (right). While K562 specific genes exhibit dose response regulation with both inhibitors, the effects are lost in the sensitive MV4;11 cell line. Similarly, specific genes that show strong dose dependence in MV4;11 cells are relatively unaffected in K562 cells. Bars represent mean ± SEM from biological replicates (n=3).

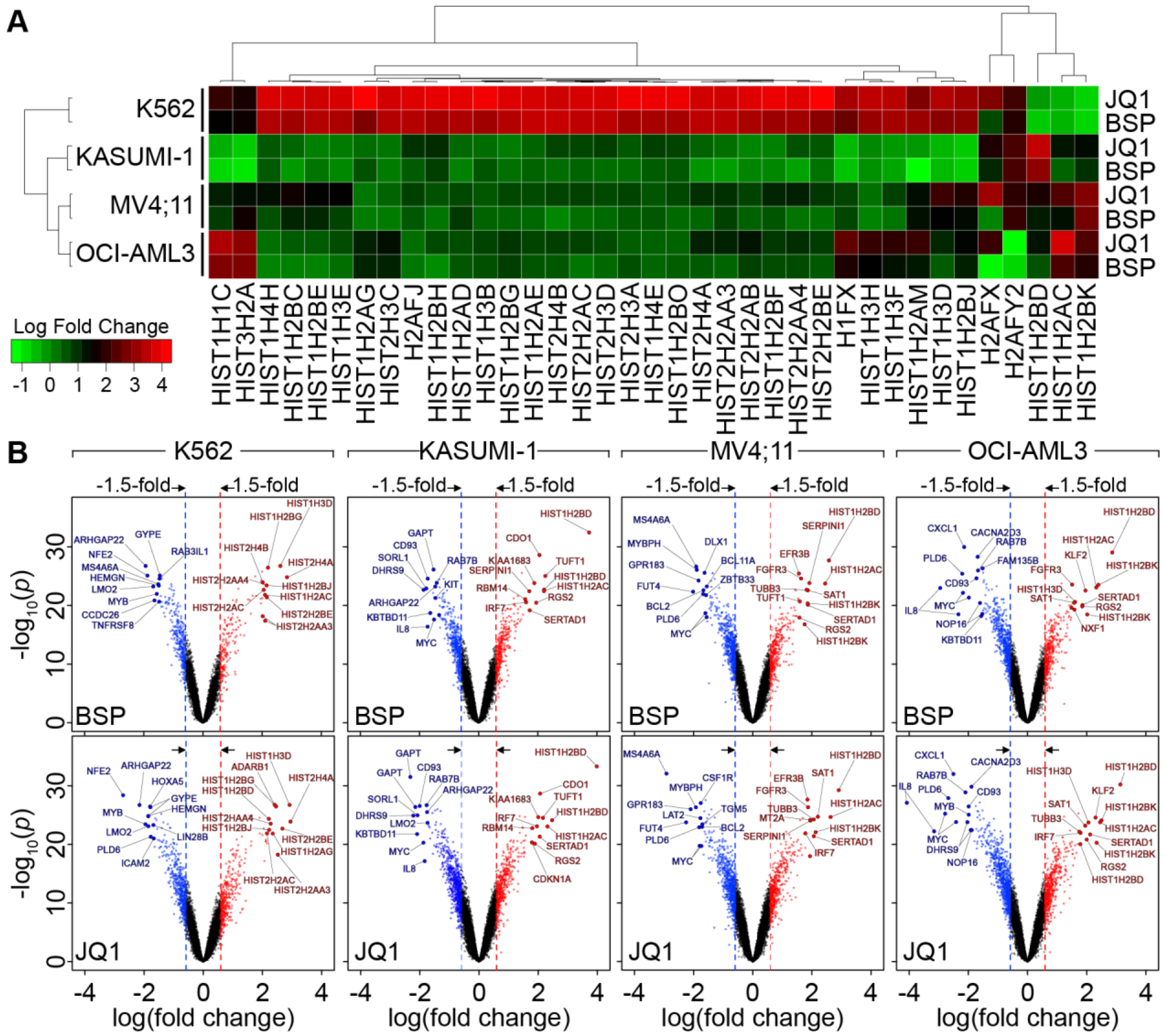

fig. S5. Effect of BSP and JQ1 on leukemia cell lines. (**A**) Histone clusters are differentially attenuate between leukemia cell lines upon treatment with either JQ1 or BSP. Most histones are significantly up-regulated in the resistant K562 cell line while they are down-regulated in the more sensitive cell lines. (**B**) Volcano plot of the top 1000 genes that are up/down regulated in the case of BSP (top panels) and JQ1 (lower panels) after 6 hours of treatment with 0.5 μM BSP or JQ1. The top 10 genes are sorted by their fold-change and are highlighted and coloured in red (up-regulated) or blue (down-regulated).

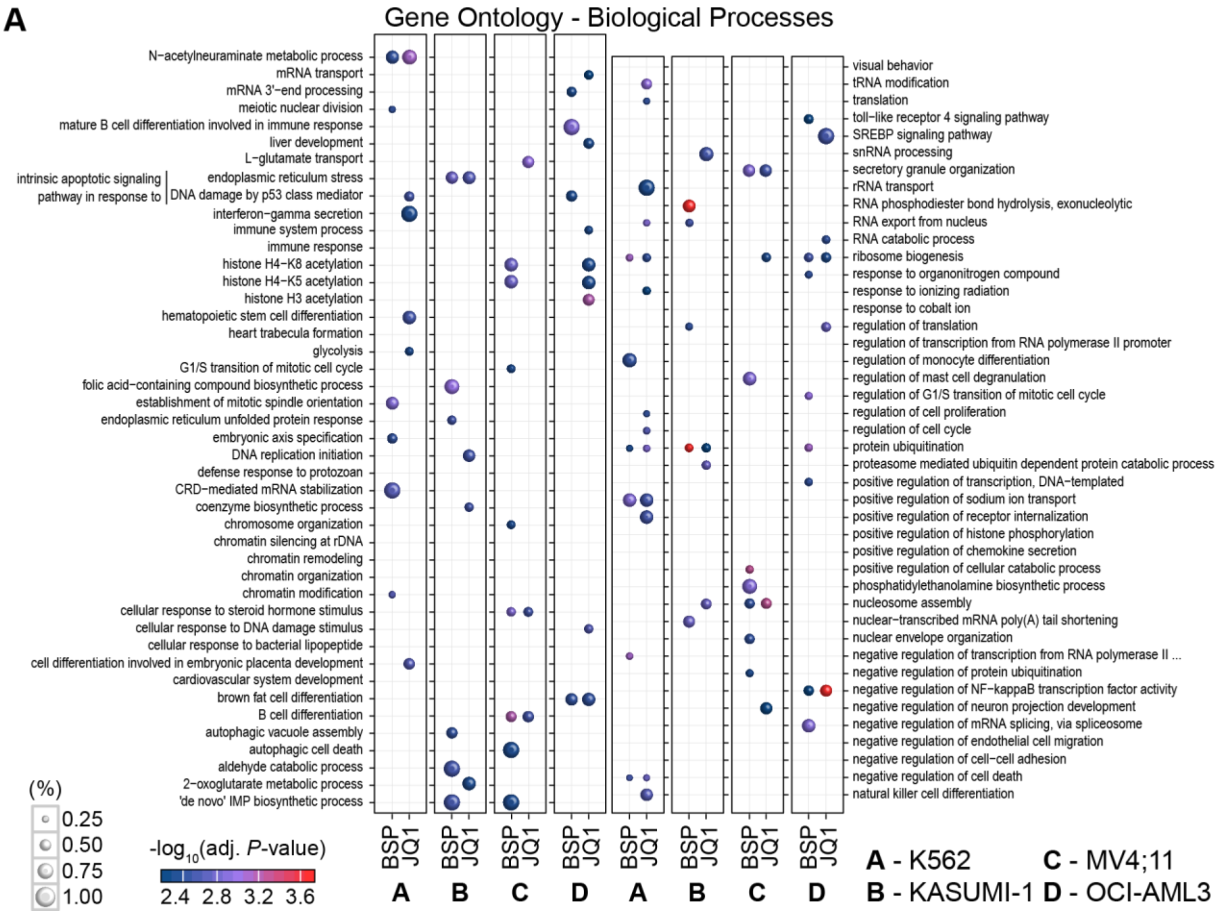

fig. S6. Gene expression GO enrichment (biological processes). Table of Gene Ontology (GO) enrichment (biological processes) for differentially expressed genes per cell line and drug treatment. Spheres represent GO term enrichment with size and color as indicated in the inset.

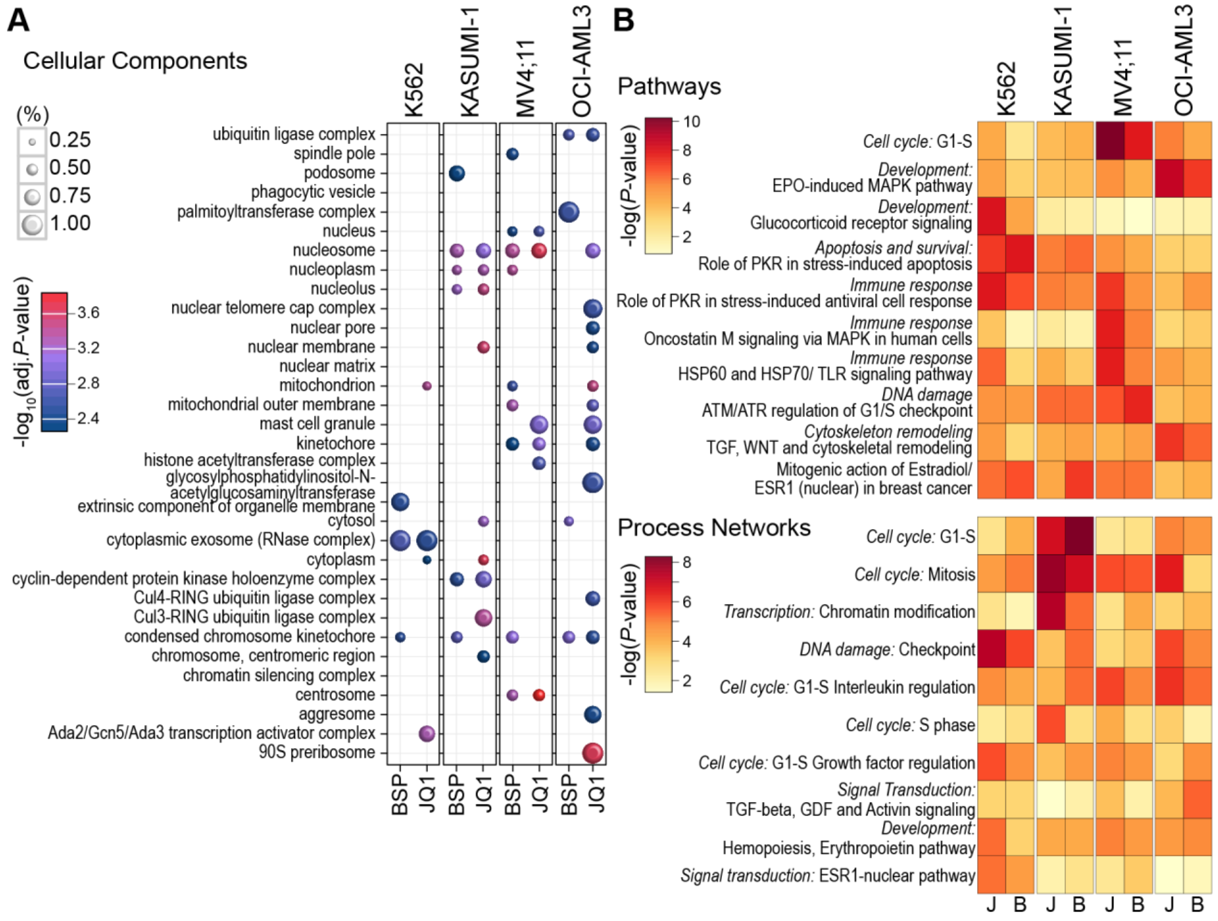

fig. S7. Gene expression after inhibition of leukemia cell lines with BSP or JQ1. (**A**) Gene Ontology (GO) enrichment of cellular components for differentially expressed genes per cell line and drug treatment. Spheres represent GO term enrichment with size and color as indicated in the inset. (**B**) MetaCore enrichment analysis for statistically significant genes identified using Benjamini-Hochberg adjusted *P*-value of < 0.001 and fold change > 1.5. *P*-values calculated from the hypergeometric intersection of significant genes with ontology entities in the MetaCore curated database are displayed for enriched pathways (upper panel) and process networks (lower panel) for the four cell lines tested. Scales are indicated in the inset. Abbreviations: J, JQ1; B, **BSP**.

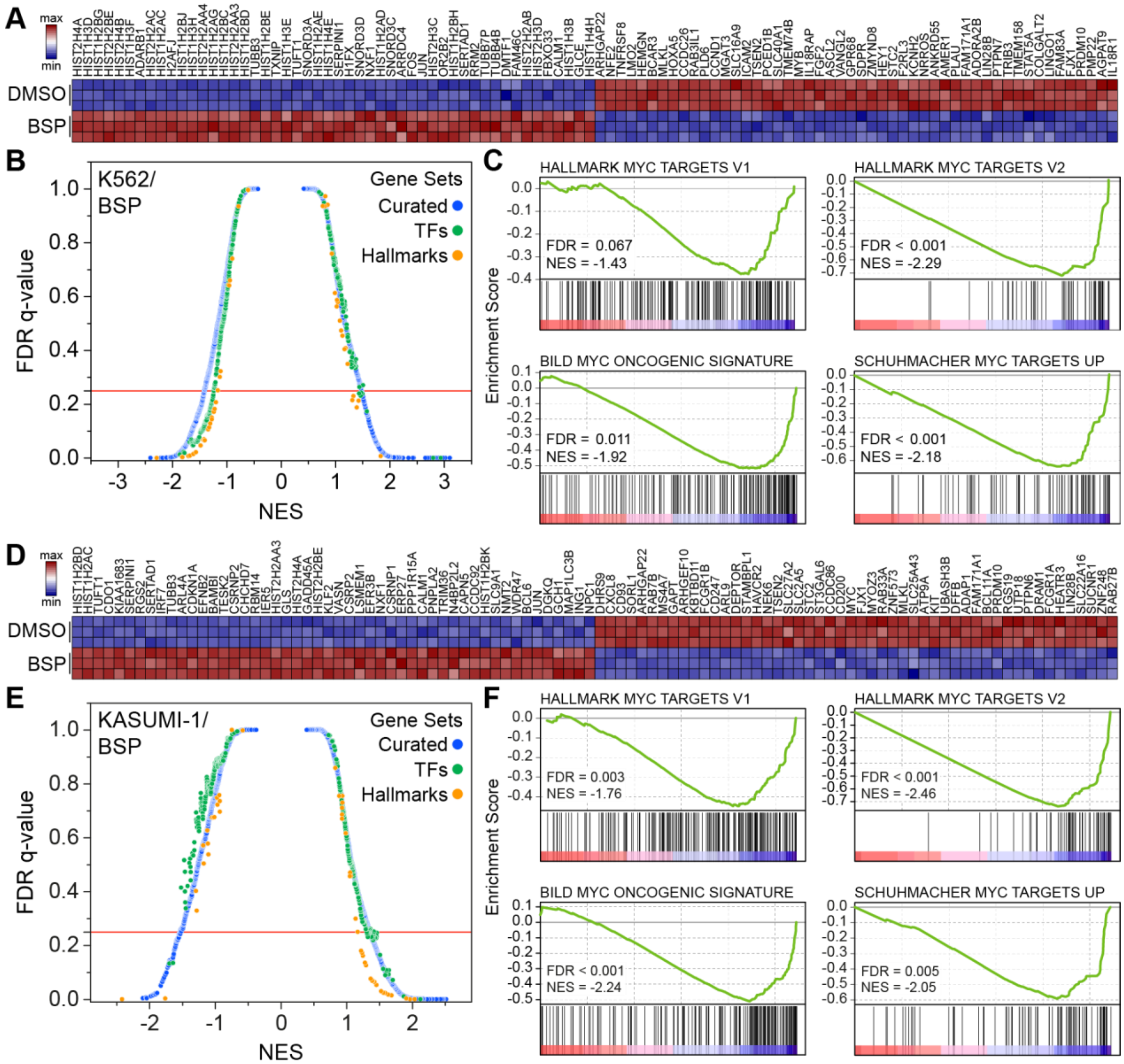

fig. S8. GSEA of K562 and KASUMI-1 cell lines after BSP treatment. (**A**) Heatmap of the top 50 up/down regulated genes in K562 cells following 6-hour BSP treatment based on 2-sided signal to noise ratio (SNR) score and $P < 0.05$. Dark blue indicates lowest expression; dark red indicates highest expression, with intermediate values represented by lighter shades, as indicated in the inset. Data are column-normalized. (**B**) Quantitative comparison of gene sets available in the MSigDB (currated (c2) in blue, transcription factors (c3) in green and hallmarks (h) in orange – MsigDB v.5.0) by GSEA for up/down regulation in BSP-treated K562 cells. Data are represented as a scatter-plot of the false discovery rate (FDR) versus the normalized enrichment score (NES) for each gene set. The red line represents the GSEA FDR cut-off (FDR q = 0.25). (**C**) GSEA demonstrating strong association with c-MYC down-regulation signatures, following 6-hour treatment of K562 cells with BSP. The plots show the running sum for the molecular signature database gene set within the K562/BSP data including the maximum enrichment score and the leading edge subset of enriched genes. (**D**) Heatmap of the top 50 up/down regulated

genes in KASUMI-1 cells following 6-hour BSP treatment based on 2-sided signal to noise ratio (SNR) score and $P < 0.05$. The heat-scale is indicated in the inset, as in (A). Data are column-normalized. (**E**) Quantitative comparison of gene sets available in the MSigDB by GSEA for up/down regulation in BSP-treated KASUMI-1 cells. Data are represented as in (B). (**F**) GSEA demonstrating strong association with c-MYC down-regulation signatures, following 6-hour treatment of KASUMI-1 cells with BSP. The plots show the running sum for the molecular signature database gene set within the KASUMI-1/BSP data including the maximum enrichment score and the leading edge subset of enriched genes.

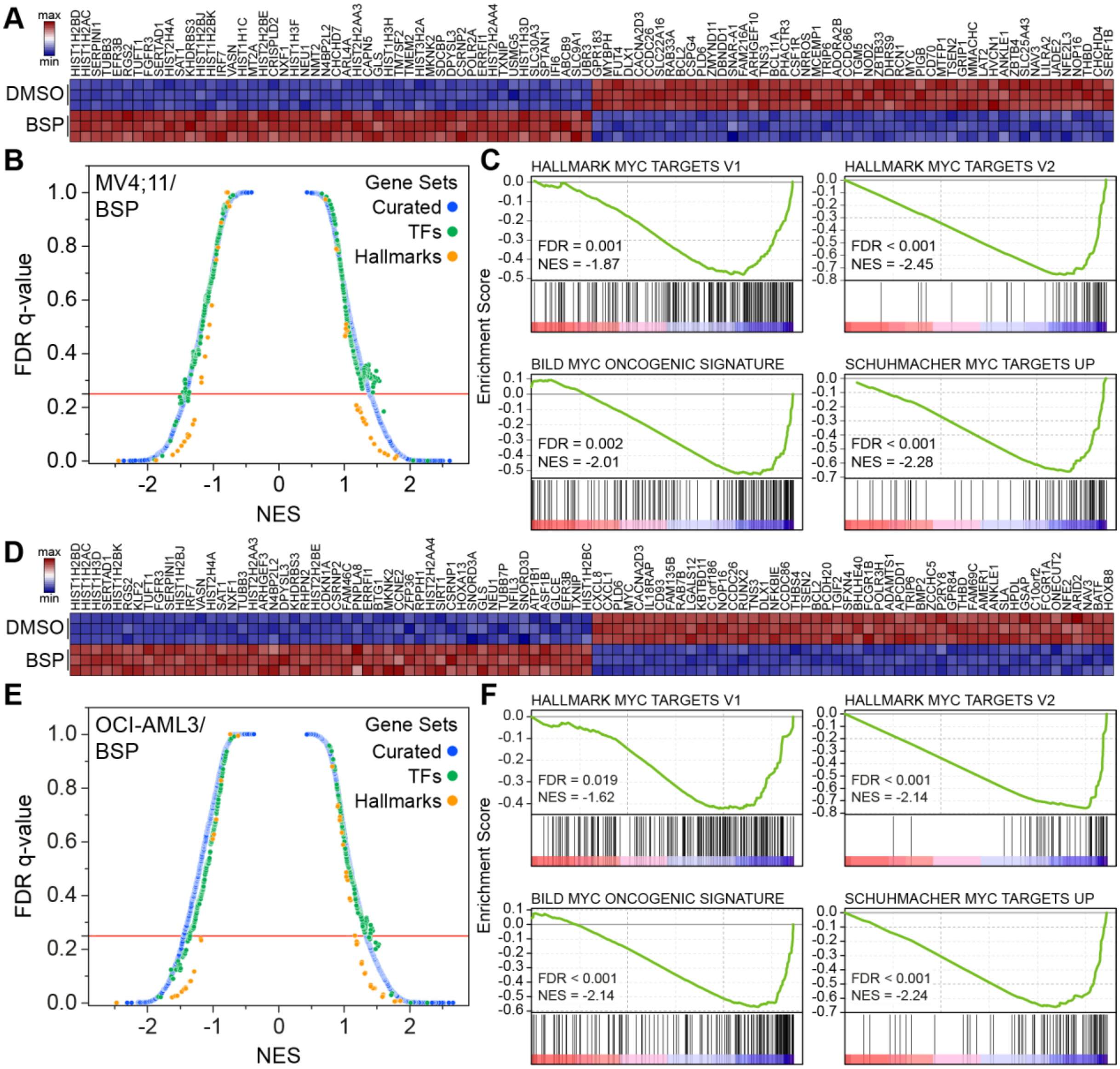

fig. S9. GSEA of MV4;11 and OCI-AML3 cell lines after BSP treatment. (**A**) Heatmap of the top 50 up/down regulated genes in MV4;11 cells following 6-hour BSP treatment based on 2-sided signal to noise ratio (SNR) score and *P* < 0.05. Dark blue indicates lowest expression, dark red indicates highest expression, with intermediate values represented by lighter shades, as indicated in the inset. Data are column-normalized. (**B**) Quantitative comparison of gene sets available in the MSigDB (currated (c2) in blue, transcription factors (c3) in green and hallmarks (h) in orange – MsigDB v.5.0) by GSEA for up/down regulation in BSP-treated MV4;11 cells. Data are represented as a scatter-plot of the false discovery rate (FDR) versus the normalized enrichment score (NES) for each gene set. The red line represents the GSEA FDR cut-off (FDR q = 0.25). (**C**) GSEA demonstrating strong association with c-MYC dependent gene-set signatures, following 6 h treatment of MV4;11 cells with BSP. The plots show the running sum for the molecular signature database gene set within the MV4;11/BSP data including the maximum enrichment score and the leading edge subset of enriched genes. (**D**) Heatmap of the

top 50 up/down regulated genes in OCI-AML3 cells following 6 hour BSP treatment based on 2-sided signal to noise ratio (SNR) score and $P < 0.05$. The heat-scale is indicated in the inset, as in (A). Data are column-normalized. (**E**) Quantitative comparison of gene sets available in the MSigDB by GSEA for up/down regulation in BSP-treated OCI-AML3 cells. Data are represented as in (B). (**F**) GSEA demonstrating strong association with c-MYC dependent gene-set signatures, following 6-hour treatment of OCI-AML3 cells with BSP. The plots show the running sum for the molecular signature database gene set within the OCI-AML3/BSP data including the maximum enrichment score and the leading edge subset of enriched genes.

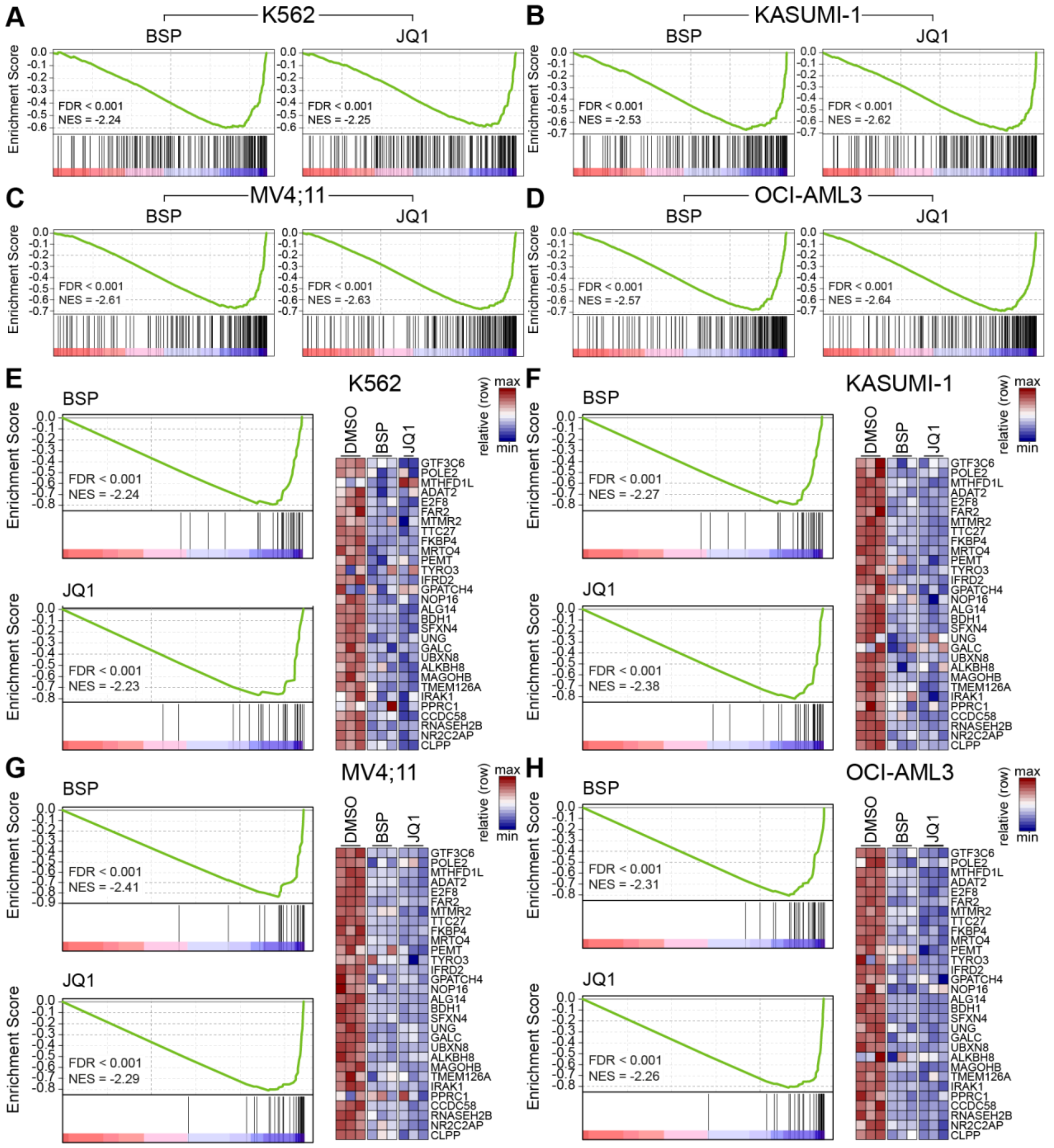

fig. S10. Effect of BSP on BET-specific genes. (**A-D**) Both BSP and JQ1 elicit a strong enrichment of a set of genes that were previously shown to be down-regulated following 24-hour treatment of THP1 cells (AML) with 250 nM of JQ1. Strong down-regulation of the same set of genes in (**A**) K562, (**B**) KASUMI-1, (**C**) MV4;11 and (**D**) OCI-AML3 is highlighted by the plots of the running sum for the molecular signature database gene set within the gene expression data of each line treated with BSP (left) or JQ1 (right) for 8 hours, including the maximum enrichment score and the leading edge subset of enriched genes. (**E-H**) Plots of the running sum of a set of genes that were previously shown to be strongly down-regulated by JQ1 in

neuroblastoma, multiple myeloma, and acute myeloid leukemia, including the maximum enrichment score and the leading edge subset of enriched genes is shown for BSP (top plots) and JQ1 (bottom plots) together with a heatmap of the top down regulated genes in (**E**) K562, (**F**) KASUMI-1, (**G**) MV4;11 and (**H**) OCI-AML3 leukemic cell lines following 6-hour treatment with 500 nM of either compound. The displayed heat maps are based on 2-sided signal to noise ratio (SNR) score and $P < 0.05$, with dark blue indicating lowest expression, dark red indicating highest expression and intermediate values represented by lighter shades. Data are row-normalized.

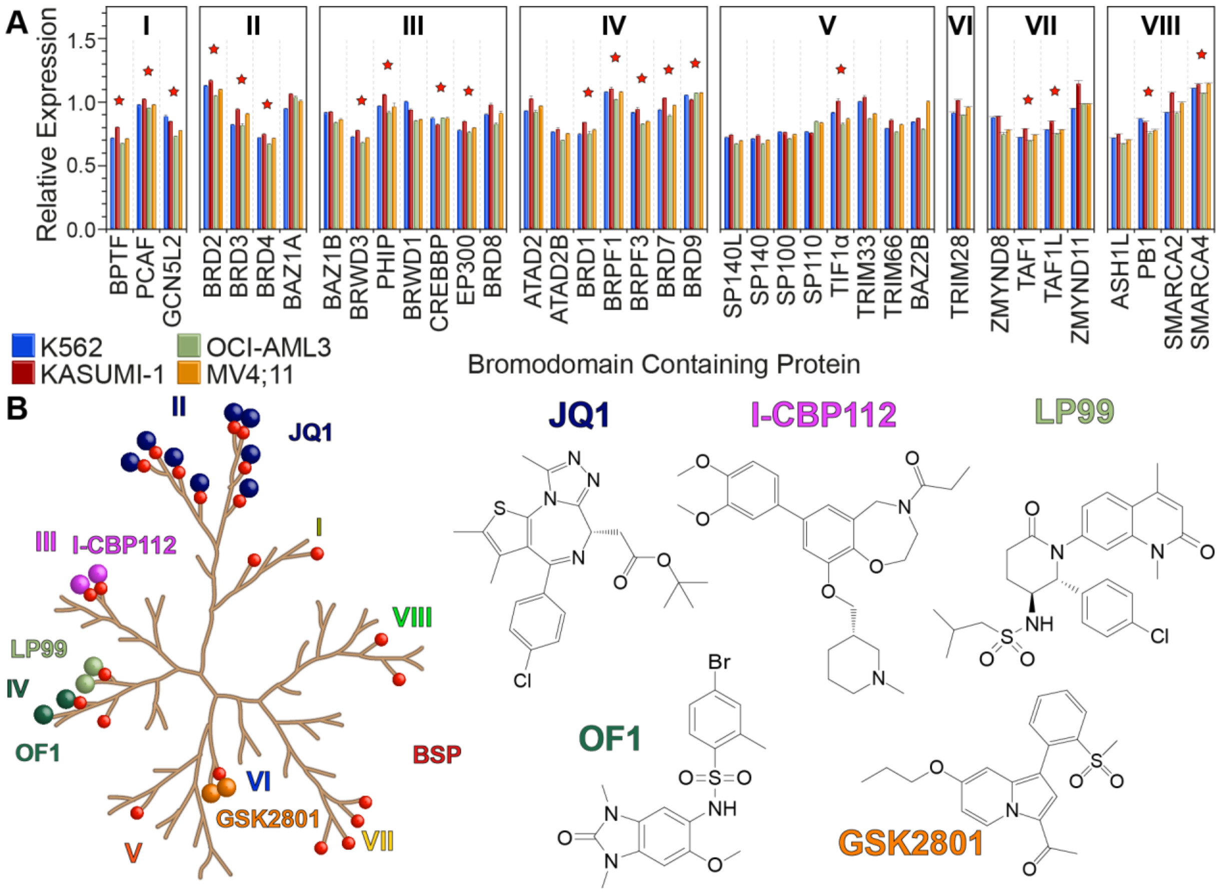

fig. S11. Expression of BRD-containing proteins in leukemic cell lines. (**A**) Relative mRNA expression of bromodomain containing proteins in K562, KASUMI-1, MV4;11 and OCI-ALM3 cells. Values are normalized against mRNA levels of SDHA in each cell line. Structural families are annotated by roman numerals and BSP target proteins are highlighted with a red star. (**B**) Selective bromodomain inhibitors targeting different sub-families (subfamily II: JQ1 shown in dark blue; III: I-CBP112 shown in magenta; IV: LP99 shown in light green and OF1 shown in dark green; V: GSK2801 shown in orange) are highlighted and their specific targets are annotated with colored spheres. In comparison, the targets of the pan-BRD inhibitor **BSP** (shown in red) are also annotated on the family tree. Structures of compounds are given on the right part of the figure.

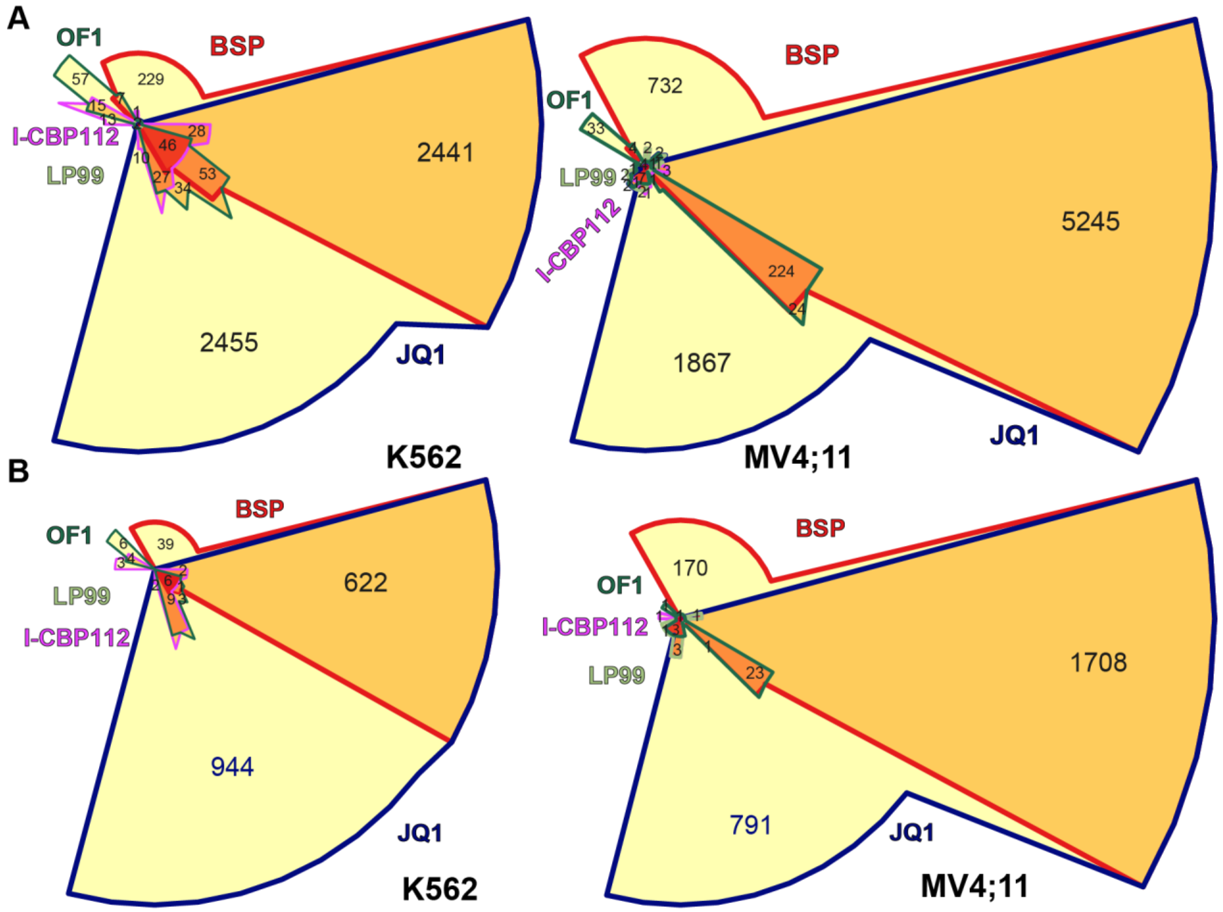

fig. S12. Effects of selective inhibition of different BRD subfamilies on transcriptional programs in leukemias. (**A**) 5-way Venn diagram of all statistically significant (using Benjamini-Hochberg adjusted *P*-value < 0.001) genes demonstrating overlap between compound treatments in K562 (left) and MV4;11 (right) cells. The majority of genes are attenuated by JQ1 however there is a number of genes that are controlled specifically by BSP with no overlap with any other inhibitors. GSK2801 had no significant effect in either cell line. (**B**) 5-way Venn diagram showing the overlap of significant (using Benjamini-Hochberg adjusted *P* < 0.001) differentially expressed genes (with fold change > 1.5) demonstrating overlap between compound treatments in K562 (left) and MV4;11 (right) cells. The majority of genes are attenuated by JQ1and BSP and only a small number are unique to BSP treatment. The effect of the other inhibitors in gene expression is negligible.

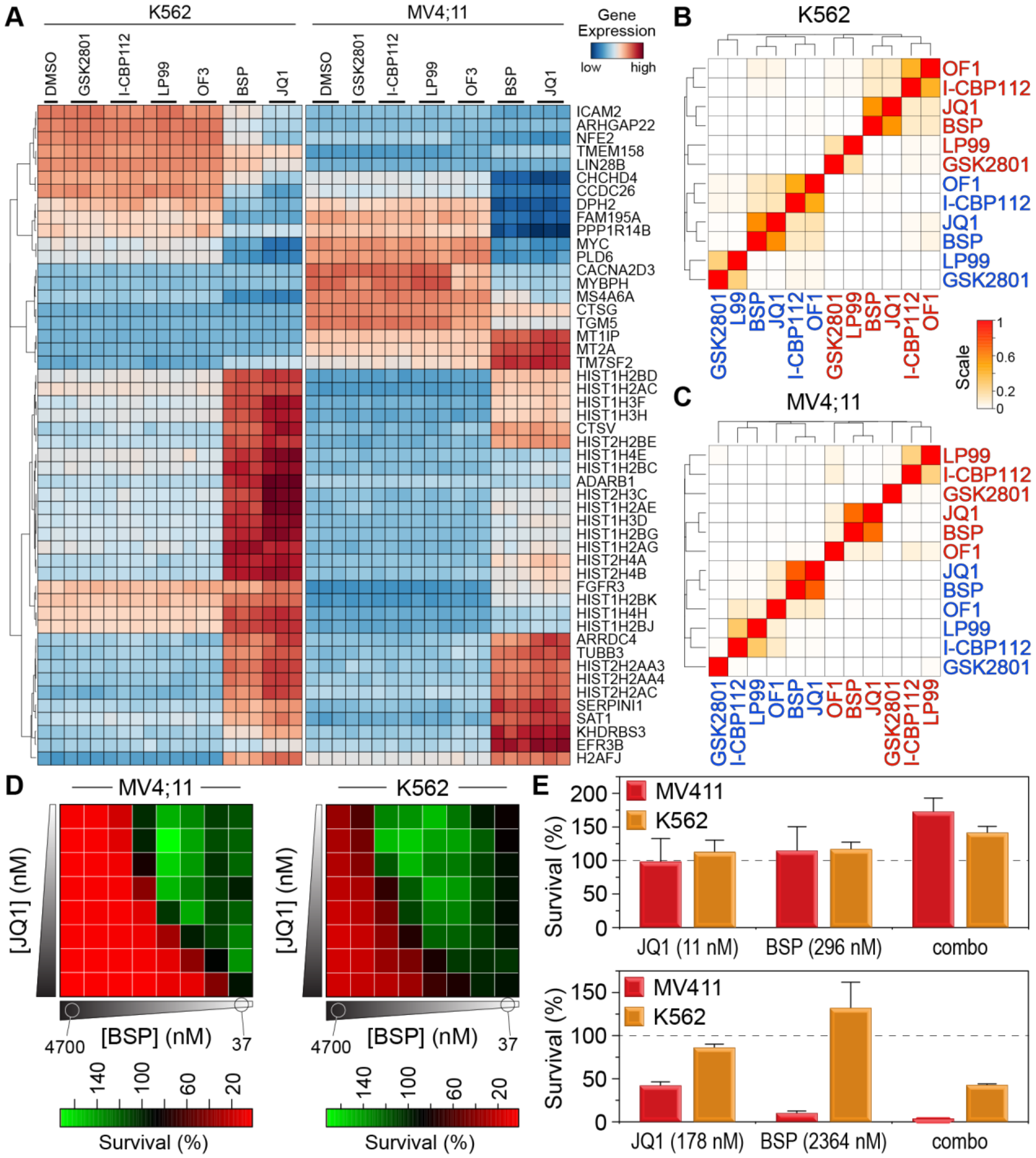

fig. S13. Transcriptional response in leukemia cell lines and inhibitor combination. Transcriptional responses to inhibition of acetylation-dependent readout in sensitive (MV4;11) and resistant (K562) leukemia cell lines are dominated by BET bromodomains. (**A**) Expression values of the top 50 statistically significant (using Benjamini-Hochberg adjusted *P*-value < 0.001) differentially expressed genes following 6-hour treatment of the cell lines with vehicle (NT) or compound. Dark blue indicates lowest expression; dark red indicates highest expression, with intermediate values represented by lighter shades. (**B**) Similarity comparison of significantly expressed genes (using Benjamini-Hochberg adjusted *P*-value < 0.001 and fold

change > 1.5) in K562 cells following 6 hour treatment with each inhibitor or vehicle. The heatmap shows the intersect matrix for all pair-wise comparisons of different compound treatments (compounds are colored so that red represents up-regulated genes and blue represents down-regulated genes per treatment) using Euclidean distances and complete linkage following transformation of the intersect counts into similarity Jaccard similarity indices. (**C**) Similarity comparison of significantly expressed genes (using Benjamini-Hochberg adjusted *P*-value < 0.001 and fold change > 1.5) in MV4;11 cells following 6-hour treatment with each inhibitor or vehicle. The heatmap shows the intersect matrix for all pair-wise comparisons of different compound treatments (compounds are colored so that red represents up-regulated genes and blue represents down-regulated genes per treatment) using euclidean distances and complete linkage following transformation of the intersect counts into similarity Jaccard similarity indices. The color scale for (B) and (C) is displayed in the inset. (**D**) Cell viability measured in a WST-1 assay within a range of BSP concentrations (37 to 4700 nM) combined with JQ1 (0.7 to 11400 nM) in MV4;11 (left) and K562 (right cells). (**E**) Combination of JQ1 and BSP bellow the EC50 value of each cell line resulted in an antagonistic effect in cell viability (top panel); combinations above EC50 values resulted in synergistic effects, mainly at concentrations of BSP above 2μM.

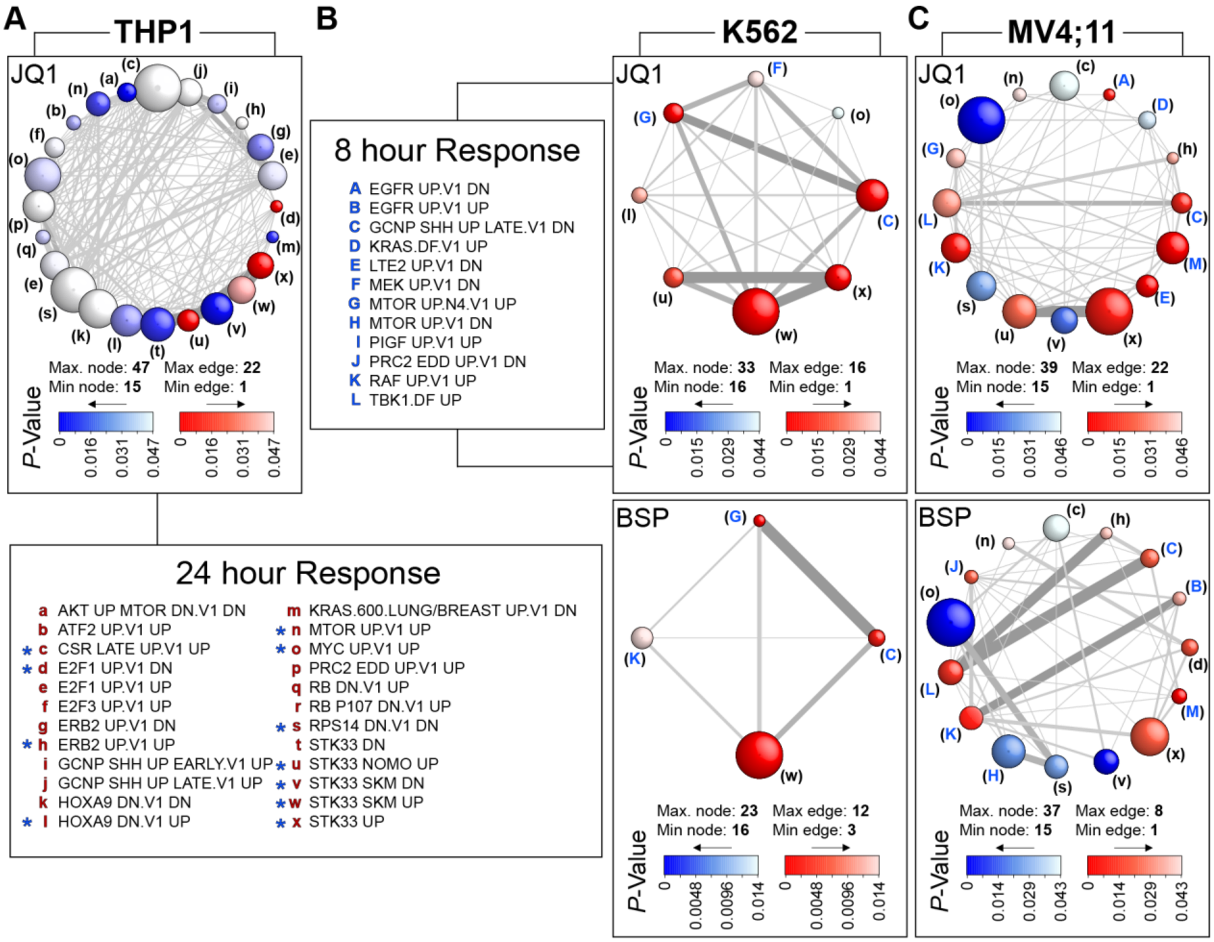

fig. S14. GSEA comparison of BSP and JQ1 effects in leukemias. (**A**) Distinct-directional network map of gene set enrichment analysis in THP1 cells treated with 250 nM JQ1 for 24 hours. Significant genes ($P < 0.001$ and fold change > 2.5) were used to determine the enrichment of gene sets found in the in the oncogenic signatures (c6) set of MSigDB. are coloured by significance (red indicating up-regulation and blue indicating down-regulation). Nodes are connected based on the genes they share by grey lines, with edge thickness correlating with the number of shared genes. (**B**) Distinct-directional network map of gene set enrichment analysis in K562 treated cells with BSP (left) or JQ1 (right) against gene sets found in the oncogenic signatures (c6) set of MSigDB. Enriched sets are represented as in (A). (**C**) Distinct-directional network map of gene set enrichment analysis in MV4;11 treated cells with BSP (left) or JQ1 (right) against gene sets found in the in the oncogenic signatures (c6) set of MSigDB. Enriched sets are represented as nodes and are coloured by significance (red indicating up-regulation and blue indicating down-regulation). Nodes are connected based on the genes they share by grey lines, with edge thickness correlating with the number of shared genes. Enriched sets are represented as in (A) and (B). Node numbering corresponds to the MSigDB gene sets given in the inset. Only differentially expressed genes with a fold change > 1.5 and $P < 0.001$ were used in the GSE analysis shown in (B) and (C). Signatures annotated with a blue star on panel (A) are also present in the 8 hour treatments in (B) and (C).

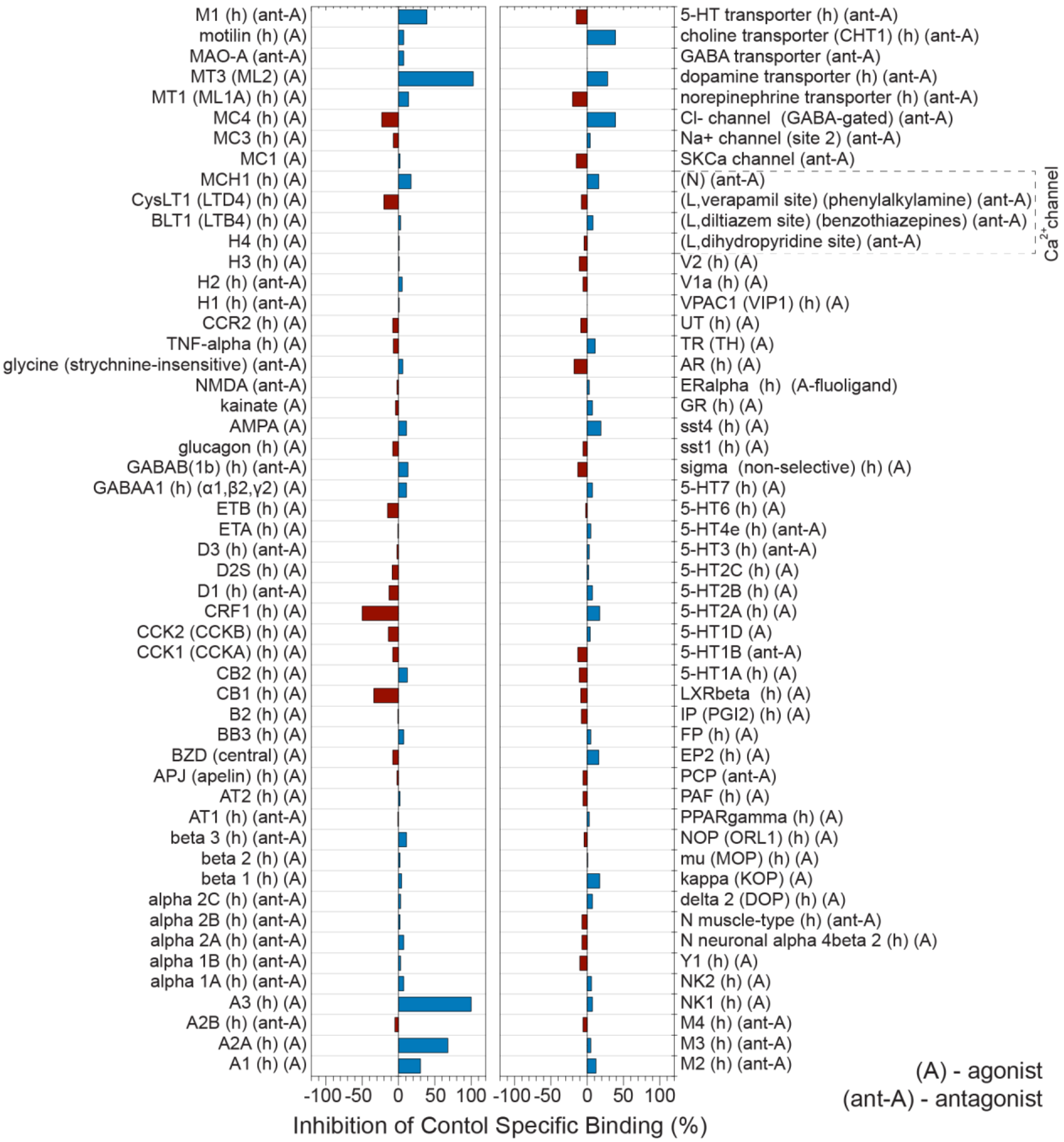

fig. S15. BSP profile of cellular receptor activity (ExpresSProfile, CEREP). Bromosporine (BSP, 10 µM) was screened against a panel of 104 ligand receptors, ion channels and transport proteins using the commercial ExpresSProfile CEREP assay. The compound exhibited no inhibitory activity toward most agonists or antagonists tested. Data represent mean and SEM of at least three independent measurements. Complete data are provided in table S6.

Supplemental Tables

table S1 (Excel Spreadsheet). Differential scanning fluorimetry profiling of triazolopyridazines against a panel of BRD modules.

table S2 (Excel Spreadsheet). MetaCore analysis of gene expression data. Genes exhibiting a differential expression upon **BSP** or JQ1 treatment (Benjamini-Hochberg adjusted. *P*-value < 0.01) were subjected to enrichment analyses in the MetaCore software suite (MetaCore from Thompson Reuters. v.6.19.65960) to identify signaling and metabolic pathways, as well as cell process networks over-represented in the differentially expressed gene sets. Statistically enriched pathways and networks were identified using a threshold FDR of 0.001.

table S3. BSP profile of cellular receptor activity data (ExpresSProfile; CEREP). Summary of BSP binding studies performed against a panel of human recombinant ligand and ion receptors. BSP (10 µM) was screened against a panel of 104 ligand receptors, ion channels and transport proteins using an established and widely utilized commercial assay (ExpresSProfile; CEREP, Paris, FRANCE).

**ASSAY**	**Catalogue Ref**	**% Inhibition of Control Specific Binding**	**1st / % of Control Specific Inhibition**	**2nd / % of Control Specific Inhibition**	**Mean / % of Control Specific Binding**	**SEM % Control**	**Reference Compound**	**IC50 Ref. (M)**	**Ki Ref. (M)**	**nH Ref.**
A1 (h) (agonist radioligand)	0442	30	66.5	72.8	69.6	3.2	CPA	1.4E-09	5.7E-10	1.1
A2A (h) (agonist radioligand)	0004	68	32.6	31.1	31.9	0.8	NECA	1.8E-08	1.5E-08	0.9
A2B (h) (antagonist radioligand)	0005	-5	100.9	108.2	104.5	3.7	NECA	1.5E-06	1.4E-06	0.7
A3 (h) (agonist radioligand)	0006	100	-0.8	1.2	0.2	1.0	IB-MECA	2.1E-10	1.3E-10	0.9
alpha 1A (h) (antagonist radioligand)	2338	7	93.6	92.5	93.1	0.6	WB 4101	4.9E-10	2.5E-10	1.8
alpha 1B (h) (antagonist radioligand)	1633	3	103.7	89.6	96.6	7.1	prazosin	1.8E-10	4.9E-11	1.5
alpha 2A (h) (antagonist radioligand)	0013	7	98.2	87.8	93.0	5.2	yohimbine	4.4E-09	2.0E-09	0.8
alpha 2B (h) (antagonist radioligand)	1344	2	89.3	107.0	98.1	8.9	yohimbine	4.8E-09	3.2E-09	0.8
alpha 2C (h) (antagonist radioligand)	0016	3	96.4	97.5	97.0	0.6	yohimbine	1.9E-09	6.0E-10	1.0
beta 1 (h) (agonist radioligand)	0018	4	90.2	102.7	96.5	6.3	atenolol	1.9E-07	1.1E-07	1.1
beta 2 (h) (agonist radioligand)	0020	2	94.1	101.6	97.8	3.8	ICI 118551	7.6E-10	2.5E-10	1.0
beta 3 (h) (antagonist	0227	11	88.0	90.0	89.0	1.0	cyanopindolol	1.8E-07	1.1E-07	0.7

radioligand)										
AT1 (h) (antagonist radioligand)	0024	-1	103.6	99.2	101.4	2.2	saralasin	5.1E-10	2.6E-10	1.0
AT2 (h) (agonist radioligand)	0026	2	84.3	111.2	97.8	13.5	angiotensin-II	1.2E-10	6.0E-11	1.1
APJ (apelin) (h) (agonist radioligand)	2154	-2	102.4	100.6	101.5	0.9	apelin-13,TFA	2.2E-10	1.9E-10	1.1
BZD (central) (agonist radioligand)	0028	-8	112.0	103.2	107.6	4.4	diazepam	9.0E-09	7.5E-09	0.9
BB3 (h) (agonist radioligand)	0472	7	86.5	99.9	93.2	6.7	Bn(6-14)	7.8E-09	4.8E-09	0.9
B2 (h) (agonist radioligand)	0033	-1	99.3	101.8	100.5	1.3	NPC 567	2.6E-08	1.3E-08	0.8
CB1 (h) (agonist radioligand)	0036	-34	113.7	153.6	133.7	20.0	CP 55940	6.2E-10	5.4E-10	0.8
CB2 (h) (agonist radioligand)	0037	12	89.3	86.3	87.8	1.5	WIN 55212-2	1.6E-09	1.0E-09	0.6
CCK1 (CCKA) (h) (agonist radioligand)	0039	-8	109.4	107.3	108.4	1.1	CCK-8s	1.4E-10	1.1E-10	1.4
CCK2 (CCKB) (h) (agonist radioligand)	0041	-14	113.8	114.3	114.1	0.3	CCK-8s	8.8E-11	3.5E-11	0.8
CRF1 (h) (agonist radioligand)	1467	-50	157.8	142.1	149.9	7.9	sauvagine	1.3E-10	8.0E-11	0.6
D1 (h) (antagonist radioligand)	0044	-13	113.0	112.3	112.7	0.4	SCH 23390	2.5E-10	1.0E-10	0.8
D2S (h) (agonist radioligand)	1322	-9	107.1	111.4	109.3	2.2	7-OH-DPAT	1.3E-09	5.1E-10	0.9
D3 (h) (antagonist radioligand)	0048	-2	104.1	99.4	101.8	2.4	(+)butaclamol	1.8E-09	4.1E-10	1.1
ETA (h) (agonist radioligand)	0054	-1	97.6	105.3	101.5	3.9	endothelin-1	2.2E-11	1.1E-11	0.8
ETB (h) (agonist radioligand)	0056	-15	114.7	115.0	114.9	0.2	endothelin-3	3.5E-11	2.0E-11	0.8
GABAA1 (h) (alpha 1,beta 2,gamma 2) (agonist radioligand)	3051	11	91.1	87.7	89.4	1.7	muscimol	9.1E-08	6.1E-08	1.1
GABAB(1b) (h) (antagonist radioligand)	0885	13	90.4	84.4	87.4	3.0	CGP 54626	1.3E-09	6.7E-10	0.8
glucagon (h) (agonist radioligand)	1407	-8	109.2	106.9	108.1	1.2	glucagon	9.7E-10	7.1E-10	0.6
AMPA (agonist radioligand)	0064	11	80.3	96.8	88.6	8.3	L-glutamate	3.6E-07	3.3E-07	0.9
kainate (agonist radioligand)	0065	-4	106.4	101.2	103.8	2.6	kainic acid	1.6E-08	1.3E-08	3.0
NMDA (antagonist radioligand)	0066	-2	111.9	91.4	101.6	10.3	CGS 19755	2.3E-07	1.9E-07	1.0
glycine (strychnine-insensitive) (antagonist radioligand)	0068	6	93.0	94.1	93.6	0.6	glycine	4.1E-07	3.7E-07	0.8
TNF-alpha (h) (agonist radioligand)	0076	-7	98.6	115.4	107.0	8.4	TNF-alpha	1.7E-10	5.5E-11	1.3
CCR2 (h) (agonist radioligand)	0362	-8	119.8	96.4	108.1	11.7	MCP-1	6.4E-11	2.6E-11	1.2
H1 (h) (antagonist radioligand)	0870	1	97.2	101.1	99.2	2.0	pyrilamine	9.1E-10	5.7E-10	1.1
H2 (h) (antagonist	1208	5	90.4	98.8	94.6	4.2	cimetidine	2.4E-07	2.4E-07	1.0

radioligand)										
H3 (h) (agonist radioligand)	1332	1	93.6	104.0	98.8	5.2	(R)alpha -Me-histamine	1.7E-09	4.2E-10	1.3
H4 (h) (agonist radioligand)	1384	1	90.7	107.5	99.1	8.4	imetit	1.2E-08	5.1E-09	0.7
BLT1 (LTB4) (h) (agonist radioligand)	1209	3	105.5	87.5	96.5	9.0	LTB4	2.2E-10	1.1E-10	1.0
CysLT1 (LTD4) (h) (agonist radioligand)	0086	-20	116.2	124.3	120.3	4.1	LTD4	2.1E-10	9.3E-11	1.2
MCH1 (h) (agonist radioligand)	1115	17	85.8	80.0	82.9	2.9	human MCH	1.4E-10	1.3E-10	0.7
MC1 (agonist radioligand)	0644	2	99.3	96.0	97.6	1.7	NDP-alpha -MSH	1.5E-10	7.6E-11	1.0
MC3 (h) (agonist radioligand)	0447	-7	109.9	105.0	107.4	2.5	NDP-alpha -MSH	3.9E-10	3.3E-10	1.3
MC4 (h) (agonist radioligand)	0420	-23	123.6	122.7	123.1	0.5	NDP-alpha -MSH	1.9E-10	1.8E-10	0.9
MT1 (ML1A) (h) (agonist radioligand)	1538	14	83.6	88.3	86.0	2.4	melatonin	2.0E-10	1.6E-10	0.9
MT3 (ML2) (agonist radioligand)	0088	103	0.4	-5.5	-2.5	3.0	melatonin	1.7E-07	1.7E-07	0.7
MAO-A (antagonist radioligand)	0443	7	93.6	91.8	92.7	0.9	clorgyline	1.2E-09	6.8E-10	1.4
motilin (h) (agonist radioligand)	0470	7	97.6	89.0	93.3	4.3	[Nleu13]-motilin	2.0E-09	1.7E-09	0.8
M1 (h) (antagonist radioligand)	0091	39	57.9	64.7	61.3	3.4	pirenzepine	1.5E-08	1.3E-08	0.9
M2 (h) (antagonist radioligand)	0093	12	88.8	87.5	88.1	0.7	methoctramine	3.2E-08	2.2E-08	1.2
M3 (h) (antagonist radioligand)	0095	5	104.9	85.7	95.3	9.6	4-DAMP	6.1E-10	4.3E-10	1.3
M4 (h) (antagonist radioligand)	0096	-6	106.8	104.6	105.7	1.1	4-DAMP	3.1E-10	1.9E-10	1.3
NK1 (h) (agonist radioligand)	0100	7	84.9	101.6	93.3	8.4	[Sar9,Met(O2)11]-SP	4.4E-10	1.9E-10	0.8
NK2 (h) (agonist radioligand)	0102	6	96.2	92.2	94.2	2.0	[Nleu10]-NKA (4-10)	2.8E-09	1.5E-09	0.8
Y1 (h) (agonist radioligand)	0106	-10	101.0	119.0	110.0	9.0	NPY	1.2E-10	8.4E-11	1.6
N neuronal alpha 4beta 2 (h) (agonist radioligand)	3029	-7	106.4	108.1	107.3	0.9	nicotine	3.0E-09	1.0E-09	0.9
N muscle-type (h) (antagonist radioligand)	0936	-7	104.2	109.8	107.0	2.8	alpha -bungarotoxin	3.2E-09	3.0E-09	0.9
delta 2 (DOP) (h) (agonist radioligand)	0114	7	96.9	88.4	92.7	4.3	DPDPE	5.3E-09	3.2E-09	1.1
kappa (KOP) (agonist radioligand)	1971	17	88.6	78.0	83.3	5.3	U 50488	5.6E-10	3.8E-10	1.0
mu (MOP) (h) (agonist radioligand)	0118	1	91.4	105.9	98.7	7.3	DAMGO	8.9E-10	3.7E-10	0.9
NOP (ORL1) (h) (agonist radioligand)	0358	-4	103.0	105.7	104.4	1.4	nociceptin	5.4E-10	1.8E-10	1.1
PPARgamma (h) (agonist radioligand)	0641	3	102.0	92.1	97.1	5.0	rosiglitazone	1.4E-08	7.6E-09	1.0
PAF (h) (agonist radioligand)	0915	-6	98.6	113.3	105.9	7.4	C16-PAF	1.5E-09	7.7E-10	1.1
PCP (antagonist	0124	-6	107.0	105.7	106.3	0.7	MK 801	1.0E-08	5.8E-09	1.1

radioligand)										
EP2 (h) (agonist radioligand)	1955	16	86.8	81.9	84.4	2.5	PGE2	3.0E-09	1.5E-09	0.9
FP (h) (agonist radioligand)	1979	5	90.6	99.6	95.1	4.5	PGF2alpha	3.4E-09	2.2E-09	1.3
IP (PGI2) (h) (agonist radioligand)	2230	-8	107.0	108.9	107.9	1.0	iloprost	2.7E-08	1.5E-08	1.0
LXRbeta (h) (agonist radioligand)	2047	-9	115.5	103.4	109.4	6.1	22(R)-hydroxycholesterol	2.4E-06	1.7E-06	1.1
5-HT1A (h) (agonist radioligand)	0131	-11	100.9	121.1	111.0	10.1	8-OH-DPAT	5.5E-10	3.4E-10	0.9
5-HT1B (antagonist radioligand)	0132	-13	115.3	110.3	112.8	2.5	serotonin	1.5E-08	9.0E-09	0.5
5-HT1D (agonist radioligand)	1974	4	91.2	101.1	96.2	5.0	serotonin	2.1E-09	7.1E-10	1.1
5-HT2A (h) (agonist radioligand)	0471	17	75.6	90.1	82.8	7.3	(±)DOI	2.6E-10	1.9E-10	1.0
5-HT2B (h) (agonist radioligand)	1333	7	91.0	95.2	93.1	2.1	(±)DOI	3.0E-09	1.5E-09	0.8
5-HT2C (h) (agonist radioligand)	1003	2	97.1	98.3	97.7	0.6	(±)DOI	8.8E-10	7.9E-10	0.9
5-HT3 (h) (antagonist radioligand)	0411	3	98.8	94.9	96.8	2.0	MDL 72222	9.8E-09	6.8E-09	0.9
5-HT4e (h) (antagonist radioligand)	0501	5	95.8	94.3	95.0	0.8	serotonin	2.4E-07	8.1E-08	0.8
5-HT6 (h) (agonist radioligand)	0142	-2	107.1	97.2	102.2	5.0	serotonin	1.4E-07	6.8E-08	0.9
5-HT7 (h) (agonist radioligand)	0144	7	87.0	98.4	92.7	5.7	serotonin	2.6E-10	9.5E-11	1.1
sigma (non-selective) (h) (agonist radioligand)	3500	-13	116.0	109.3	112.6	3.4	haloperidol	7.8E-08	6.2E-08	0.9
sst1 (h) (agonist radioligand)	1940	-6	108.3	103.0	105.6	2.7	somatostatin-28	3.4E-10	3.1E-10	0.7
sst4 (h) (agonist radioligand)	0482	19	83.7	78.2	81.0	2.8	somatostatin-14	3.8E-09	3.8E-09	0.6
GR (h) (agonist radioligand)	0469	7	90.4	95.2	92.8	2.4	dexamethasone	3.3E-09	1.6E-09	1.3
ERalpha (h) (agonist fluoligand)	0484	3	94.8	98.6	96.7	1.9	17-beta -estradiol	9.1E-09	7.3E-09	5.0
AR (h) (agonist radioligand)	0933	-18	115.8	120.3	118.1	2.3	mibolerone	2.0E-09	9.1E-10	1.2
TR (TH) (agonist radioligand)	0156	11	87.8	89.8	88.8	1.0	T3	4.9E-10	3.4E-10	0.9
UT (h) (agonist radioligand)	1386	-9	113.7	103.3	108.5	5.2	urotensin-II	6.1E-10	4.6E-10	1.5
VPAC1 (VIP1) (h) (agonist radioligand)	0157	0	100.0	100.0	100.0	0.0	VIP	1.9E-10	1.1E-10	2.4
V1a (h) (agonist radioligand)	0159	-6	101.0	111.8	106.4	5.4	[d(CH2)51,Tyr(Me)2]-AVP	5.5E-10	3.4E-10	0.8
V2 (h) (agonist radioligand)	0497	-11	115.6	105.9	110.7	4.9	AVP	8.9E-10	6.4E-10	1.1
Ca2+ channel (L, dihydropyridine site) (antagonist radioligand)	0161	-4	102.2	106.7	104.5	2.3	nitrendipine	3.0E-10	2.0E-10	1.0
Ca2+ channel (L, diltiazem site)	0162	8	86.3	98.0	92.2	5.9	diltiazem	5.4E-08	4.2E-08	0.7

(benzothiazepines) (antagonist radioligand)										
Ca2+ channel (L, verapamil site) (phenylalkylamine) (antagonist radioligand)	0163	-8	107.1	108.0	107.6	0.5	D 600	1.9E-08	9.3E-09	0.6
Ca2+ channel (N) (antagonist radioligand)	0164	16	82.5	84.8	83.6	1.2	omega -conotoxin GVIA	2.4E-12	9.7E-13	1.6
SKCa channel (antagonist radioligand)	0167	-15	108.3	122.4	115.3	7.1	apamin	1.3E-11	6.6E-12	1.0
Na+ channel (site 2) (antagonist radioligand)	0169	4	97.1	95.2	96.2	1.0	veratridine	6.4E-06	5.7E-06	1.1
Cl- channel (GABA-gated) (antagonist radioligand)	0170	39	59.6	62.1	60.9	1.3	picrotoxinin	1.5E-07	1.2E-07	0.9
norepinephrine transporter (h) (antagonist radioligand)	0355	-20	128.0	112.6	120.3	7.7	protriptyline	3.2E-09	2.4E-09	1.2
dopamine transporter (h) (antagonist radioligand)	0052	28	71.6	72.9	72.2	0.7	BTCP	1.1E-08	5.8E-09	1.3
GABA transporter (antagonist radioligand)	0060	0	105.6	94.1	99.8	5.8	nipecotic acid	1.6E-06	1.6E-06	0.6
choline transporter (CHT1) (h) (antagonist radioligand)	1552	39	51.6	69.5	60.6	9.0	hemicholinium-3	4.9E-09	2.8E-09	1.0
5-HT transporter (h) (antagonist radioligand)	0439	-15	112.5	116.6	114.6	2.1	imipramine	1.9E-09	8.6E-10	1.1

table S4. Data collection and refinement statistics for BRD-BSP complexes.

**Data Collection**			
**PDB ID**	**5IGK**	**5IGL**	**5IGM**
Protein/Ligand	BRD4(1)/**BSP**	TAF1L(2)/**BSP**	BRD9/**BSP**
Space group	$P2_12_12_1$	$I2_12_12_1$	$P2_12_12_1$
Cell dimensions: a, b, c (Å)<br>α, β, γ (deg)	39.47 43.85 78.02<br>90.00 90.00 90.00	44.17 89.18 117.89<br>90.00 90.00 90.00	70.18 125.68 29.79<br>90.00 90.00 90.00
Resolution* (Å)	1.70 (1.79-1.70)	2.10 (2.21-2.10)	1.60 (1.69-1.60)
Unique observations*	15007 (2101)	13934 (2005)	35807 (5105)
Completeness* (%)	97.1 (94.9.)	99.6 (99.7)	99.9 (100.0)
Redundancy*	4.7 (4.6)	3.6 (3.5)	6.4 (6.6)
Rmerge*	0.043 (0.244)	0.036 (0.369)	0.029 (0.503)
I/ σI*	21.1 (5.9)	16.6 (2.7)	28.0 (3.9)
**Refinement**			
Resolution (Å)	1.70	2.10	1.60
R_{work} / R_{free} (%)	15.6/19.9	21.1/29.4	22.2/26.6
Number of atoms (protein/other/water)	1056/32/118	1095/28/25	1833/44/120
B-factors (Å^2) (protein/other/water)21.85	21.85/15.67/29.43	69.64/74.58/58.06	37.95/42.12/35.52
r.m.s.d bonds (Å)<br>r.m.s.d angles (º)	0.014<br>1.637	0.025<br>2.337	0.014<br>1.423
Ramachadran Favoured (%)<br>Allowed (%)<br>Disallowed (%)	97.60<br>2.40<br>0.00	93.43<br>6.57<br>0.00	99.55<br>0.45<br>0.00

* Values in parentheses correspond to the highest resolution shell.

table S5. Primers used for qRT-PCR.

Gene	NCBI	Sequence (5’ →3’)		Tm (˚C)	Location	Transcript Length
CXCL8	NM_000584	fwd	CCAAGAATCAGTGAAGATGC	55.8	476-495	85
		rvs	GCAACCCTACAACAGACC	53.7	560-543	
CSF1R	NM_005211	fwd	GTTTGGTAAGACCCTCGGA	57.6	2041-2059	95
		rvs	CAGCCACCTTCAGGACA	56.5	2135-2119	
FES	NM_001143785	fwd	GCTACTCCTCCGAAAGCGA	60.6	1942-1960	106
		rvs	TCCCGTGTCTGCTGATT	55.9	2047-2031	
BCL2	NM_000633	fwd	GATGTGATGCCTCTGCG	57.1	5341-5357	81
		rvs	CTCTGGAATCTAAAGGTCGT	53.6	5421-5402	
CDK20	NM_012119	fwd	GCTAAGGTGGCATTGTCT	53.6	1850-1867	114
		rvs	GAGTGCTCAGTGATGTGAAGTA	55.6	1963-1942	
RHOU	NM_021205	fwd	GCTGTTAGGGCTGAATCTT	54.6	2886-2904	90
		rvs	CCTTGTGGTGTCTCGGA	56.3	2975-2959	
RAB33A	NM_004794	fwd	CCCAAAGAGAGCCAGAAC	55.8	817-834	87
		rvs	AGCATCACGATACAGCAG	52.9	903-886	
GATA1	NM_002049	fwd	CTGTCCCCAATAGTGCTTATGG	60.2	470-491	88
		rvs	GAATAGGCTGCTGAATTGAGGG	60.8	557-536	
FGF2	NM_002006	fwd	AGAAGAGCGACCCTCACATCA	62.7	560-580	82
		rvs	CGGTTAGCACACACTCCTTTG	61.2	641-621	
FOXA3	NM_004497	fwd	GAGATGCCGAAGGGGTATCG	61.9	310-329	164
		rvs	TGATTCTCCCGGTAGTAAGGG	60.1	473-453	
HOXA5	NM_019102	fwd	AACTCATTTTGCGGTCGCTAT	60.4	19-39	89
		rvs	TCCCTGAATTGCTCGCTCAC	62.2	107-88	
BCL6B	NM_181844	fwd	ACCCACCTACTGAATCTCGAA	60.0	506-526	112
		rvs	GCCTGAGAGTTTAGCACGATGT	62.3	617-596	
SPRY1	NM_199327	fwd	GCAGTGGCAGTTCGTTAGTTG	61.8	23-43	87
		rvs	CAGTAGGCTGAATCTCTCTCTCA	60.4	109-87	

HEY1	NM_003806	fwd	GTTCGGCTCTAGGTTCCATGT	61.5	98-118	88
		rvs	CGTCGGCGCTTCTCAATTATTC	61.9	185-164	
HEMGN	NM_018437	fwd	GTACTATGACCCGACGGATG	60.4	132-153	219
		rvs	GAGATGTCTGTCTGGGCTAG	61.1	350-330	
SMAGP	NM_001031628	fwd	ACCAGCCTCCTGACTACTCC	62.2	4-23	50
		rvs	GGGGTGGTCATCAGTTCTTCT	61.1	53-33	
18S rRNA	U13369.1	fwd	GCTTAATTTGACTCAACACGGGA	62.5	4892-4914	69
		rvs	AGCTATCAATCTGTCAATCCTGTC	58.8	4960-4937	
SDHA	NM_004168	fwd	GCATTTCAGAGACAGCCAT	56.3	1278-1296	115
		rvs	TGCCCCTTGTAGTTGGT	54.7	1392-1376	
**fwd**: forward primer; **rvs**: reverse primer						
